# Supplementary material for: Stylet cuticular gene-directed mutagenesis impairs the pea aphid vector capacity to transmit a plant virus
Source: PLoS Pathog. 2025 May 23;21(5):e1013192. doi: 10.1371/journal.ppat.1013192 (PMC12140417; doi:10.1371/journal.ppat.1013192)
Supplement: S1 Text — Setup complementary information for the analysis of aphid feeding behavior. (PDF) [file ppat.1013192.s001.pdf]

Supplementary Material and Methods

Setup complementary information for the analysis of aphid feeding behavior

Six aphids (2 per line) were connected almost simultaneously at each stage (i.e. there was only a few seconds' delay between individuals in the start of the recordings). The recordings were performed in daylight inside a Faraday cage under constant temperature (23±1 °C). A thin gold wire (Ø 10 µm, 2–3 cm long) was fixed using a drop of water-based silver glue to the thorax cuticle of the backside of the N2 that were held stationary at the tip of a plastic pipe in which a slight suction was applied. The other end of the gold wire was then inserted into the EPG head stage amplifier. A second copper rod electrode was inserted into the soil of each potted plant to close the electrical circuit. The electrical signals between the electrodes were converted into digital signals via the Di710-UL (DATAQ) analog-to-digital board. The digital signals were visualized and recorded on a computer using Probe 3.5 software (EPG Systems, Wageningen, The Netherlands). Each insect's recording signal was adjusted individually according to the 4 steps of the procedure described in the Giga-4/8 Manual to provide optimal resolution. In particular, each recording signal was adjusted individually in order that baseline was 0 V and extracellular stylet location waveform was 3–4 V.
