# Supplementary material for: Stylet cuticular gene-directed mutagenesis impairs the pea aphid vector capacity to transmit a plant virus
Source: PLoS Pathog. 2025 May 23;21(5):e1013192. doi: 10.1371/journal.ppat.1013192 (PMC12140417; doi:10.1371/journal.ppat.1013192)
Supplement: S1 Fig — Conserved amino acids are highlighted with a red background color. Predicted signal peptide sequences are underlined in black. The WT RR-1 chitin-binding domain is indicated with a double blue arrow. The multiple alignment of amino acid sequences was performed using Clustal2.1 (http://www.clustal.org/clustal2/). Alignment results were presented by ESPript 3.0 (https://espript.ibcp.fr/ESPript/ESPript/index.php). The signal peptides were predicted using the SignalP 6.0 server (https://services.healthtech.dtu.dk/services/SignalP-6.0/). The prediction and localization of chitin-binding domain were conducted in cuticleDB (http://bioinformatics2.biol.uoa.gr/cuticleDB/index.jsp). (PDF) [file ppat.1013192.s002.pdf]

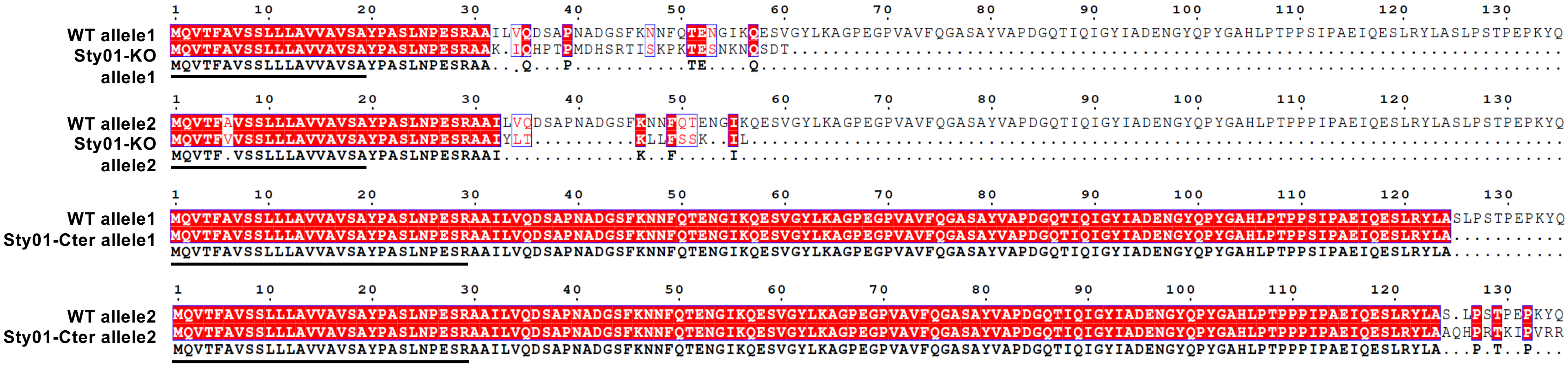

WT allele1
Sty01-KO allele1
WT allele2
Sty01-KO allele2
WT allele1
Sty01-Cter allele1
WT allele2
Sty01-Cter allele2
