# Supplementary figures and images for: Stylet cuticular gene-directed mutagenesis impairs the pea aphid vector capacity to transmit a plant virus
Source: PLoS Pathog. 2025 May 23;21(5):e1013192. doi: 10.1371/journal.ppat.1013192 (PMC12140417; doi:10.1371/journal.ppat.1013192)

**A**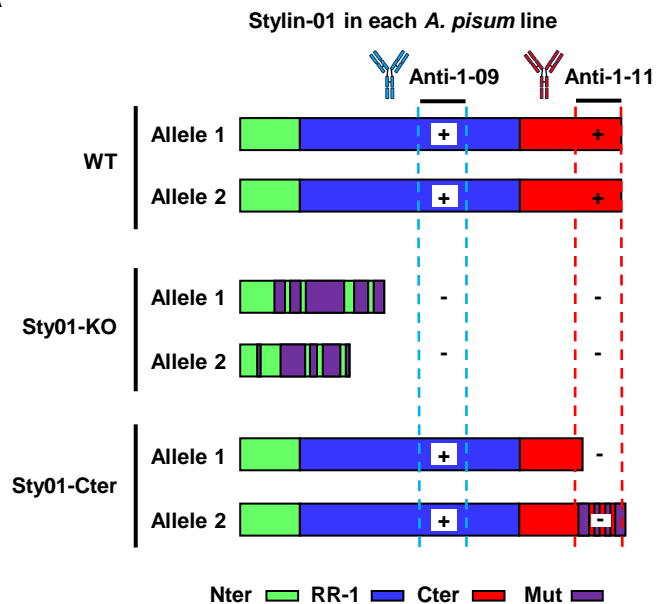**B**Stylin-01 in each *A. pisum* line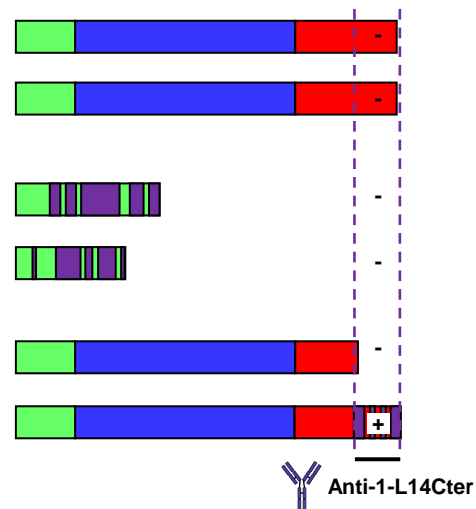**C**Stylin-02 in the three *A. pisum* lines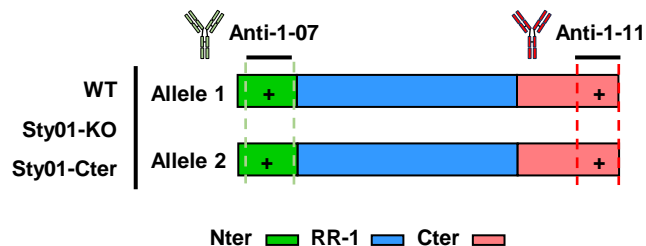

Supplement: S2 Fig — (A-C) Schematic representation of the domains of the mature Stylin-01 and Stylin-02 proteins as represented in Figure 1 with peptides targeted by the different antibodies indicated as black lines. (A) Peptides targeted by anti-1–09 or anti-1–11 antibodies initially produced in Webster et al., 2018 [14] to detect wild-type Stylin-01 protein. (B) Peptide targeted by anti-1-L14Cter specifically produced in this study to detect the mutated C-terminus of the protein encoded by the Sty01-Cter allele 2 of stylin-01 gene. (C) Peptides of Stylin-02 targeted by anti-1–07 or anti-1–11 antibodies. (+) and (-) respectively indicate whether one protein should be detected or not by the antibody, respectively. The N-terminal (Nter) and C-terminal (Cter) domains surrounding the RR-1 chitin-binding-domain are indicated in green, red and blue respectively. Mutations in the amino acid sequences (Mut) are indicated in purple. (PDF) [file ppat.1013192.s003.pdf]

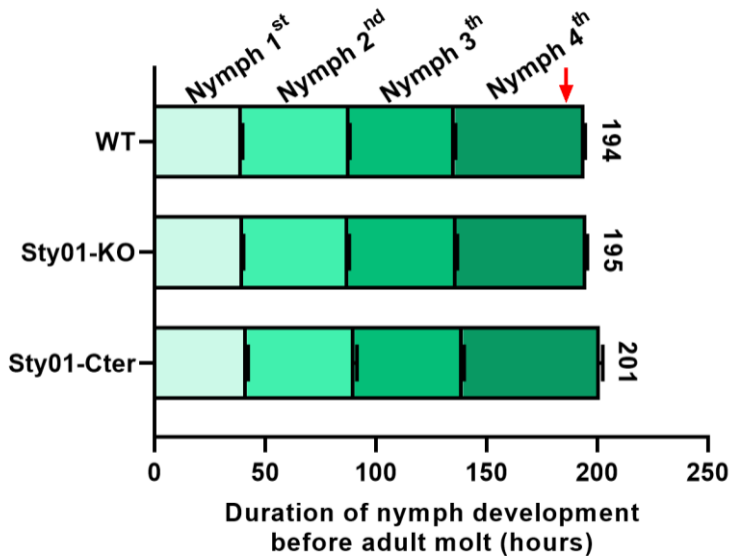

Supplement: S3 Fig — Nymphal development of WT, Sty01-KO and Sty01-Cter aphid lines at 18°C under 16:8 h (day:night) on faba beans. The diagram results from two independent biological replicates and at least 20 aphid nymphs examined per aphid line. The sampling time performed at late N4 stage 46 h post-molt is indicated by a red arrow. (PDF) [file ppat.1013192.s004.pdf]

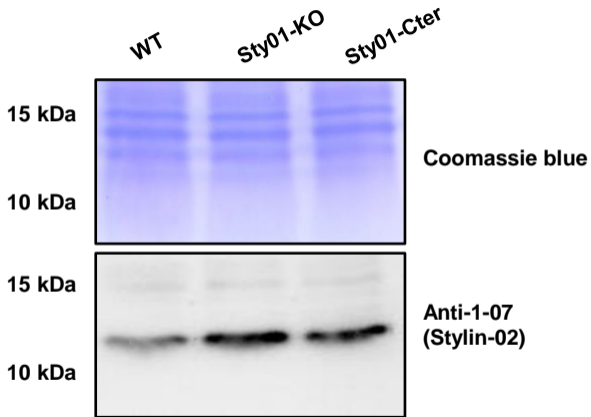

Supplement: S4 Fig — Each vertical lane corresponds to the protein crude extracts of 10 aphid whole bodies. Whole-body crude extracts proteins from the WT (lane 1), Sty01-KO (lane 2), and Sty01-Cter (lane 3) were analyzed by SDS-PAGE and stained with Coomassie blue to compare the protein amount loaded on the gel, or transferred onto a nitrocellulose membrane prior to immunolabeling with anti-1–07 antibody targeting Stylin-02 (S2C Fig and S1 Table). (PDF) [file ppat.1013192.s005.pdf]

A

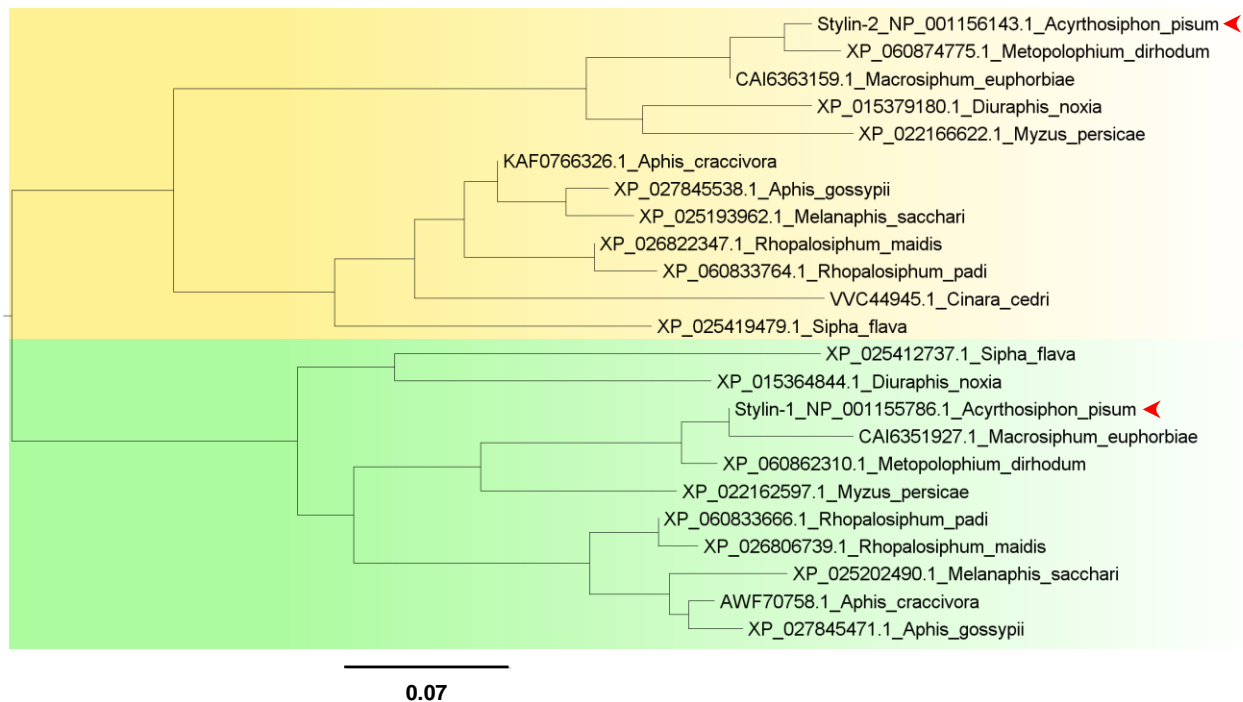

B

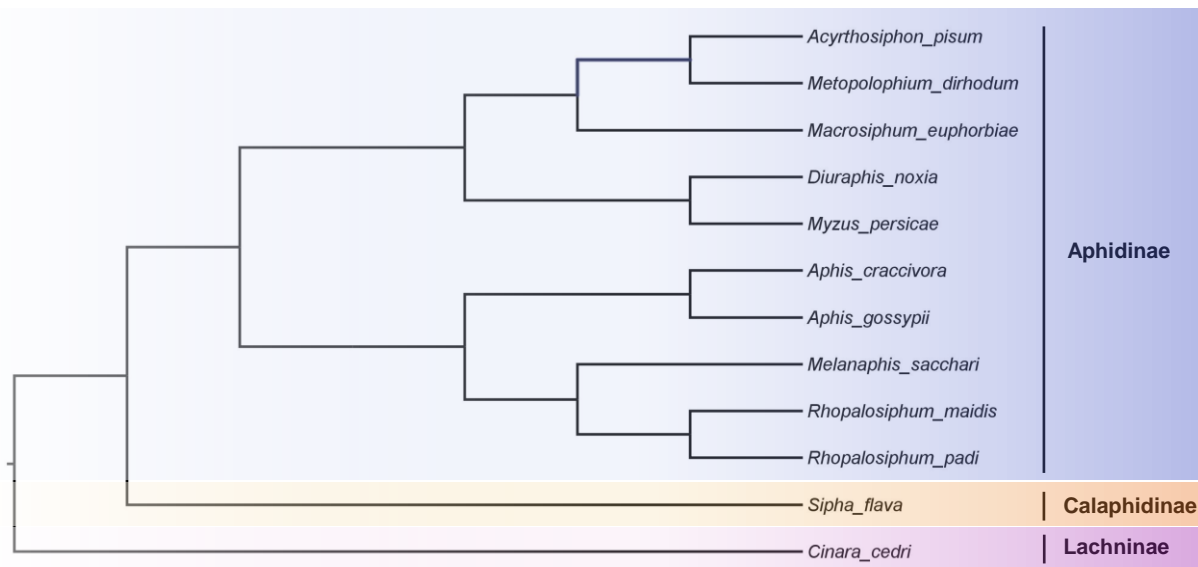

Supplement: S5 Fig — (A) Phylogenetic tree generated with IQtree based on a MAFFT alignment and edited in FigTree. Acyrthosiphon pisum Stylin-01 and Stylin-02 are indicated by a red arrow. Green backgroup for the cluster containing Stylin-01 and yellow background for the cluster containing Stylin-02. (B) The species consensus tree is based on Jousselin et al., 2024 [26] and Hardy et al., 2022 [27] of the Aphididae species for which we retrieved orthologs. (PDF) [file ppat.1013192.s006.pdf]
