# Supplementary material for: Stylet cuticular gene-directed mutagenesis impairs the pea aphid vector capacity to transmit a plant virus
Source: PLoS Pathog. 2025 May 23;21(5):e1013192. doi: 10.1371/journal.ppat.1013192 (PMC12140417; doi:10.1371/journal.ppat.1013192)
Supplement: S1 Table — (PDF) [file ppat.1013192.s007.pdf]

S1 Table. Antibodies used in the study.

Antibody ID	Peptide Sequence	Target protein	Purpose	Reference
**Anti-1-07**	SQEQEVNFDGNFKNK	Stylin-02	WB, IF	[14]
**Anti-1-09**	EVRYLKAGPEGPVSV	Stylin-01	WB	[14]
**Anti-1-11**	RYLASLPSTPEPKYQ	Stylin-01/02	IF	[14]
**Anti-1-L14Cter**	LAAQHPRTKIPVRR	Stylin-01 isoform encoded by Sty01-Cter allele 2	WB, IF	This work
**Anti-1-15**	VEGGYSYTAPDGTPI	Stylin-03	IF	[15]
**Anti-1-16**	FNGFRPNGAYPQQYI	Stylin-04/04bis	IF	[15]

WB, western blot; IF, immunofluorescence

14. Webster CG, Pichon E, van Munster M, Monsion B, Deshoux M, Gargani D, et al. Identification of Plant Virus Receptor Candidates in the Stylets of Their Aphid Vectors. J Virol. 2018;92: e00432-18. doi:10.1128/JVI.00432-18

15. Deshoux M, Masson V, Arafah K, Voisin S, Guschinskaya N, van Munster M, et al. Cuticular Structure Proteomics in the Pea Aphid Acyrthosiphon pisum Reveals New Plant Virus Receptor Candidates at the Tip of Maxillary Stylets. J Proteome Res. 2020/01/29 ed. 2020;19: 1319–1337. doi:10.1021/acs.jproteome.9b00851
