# Supplementary material for: Stylet cuticular gene-directed mutagenesis impairs the pea aphid vector capacity to transmit a plant virus
Source: PLoS Pathog. 2025 May 23;21(5):e1013192. doi: 10.1371/journal.ppat.1013192 (PMC12140417; doi:10.1371/journal.ppat.1013192)
Supplement: S2 Table — Results are expressed as mean hours (± SE) of at least 20 nymphs per aphid line. (PDF) [file ppat.1013192.s008.pdf]

S2 Table. Duration of the development of wild-type and mutant nymphs before adult molt at 18 °C and a 16/8 h (day/night) photoperiod. Results are expressed as mean hours (± SE) of at least 20 nymphs per aphid line

	Nymph stage				
**Aphid lines**	**Nymph 1 (N1)**	**Nymph 2 (N2)**	**Nymph 3 (N3)**	**Nymph 4 (N4)**	**Total duration**
**WT**	39.6±0.2 a	48.4±0.3 a	47.9±0.3 a	58.5±0.5 a	194.3±0.7 a
**Sty01-KO**	39.9±0.4 ab	47.7±0.4 a	48.9±0.4 ab	58.7±0.5 a	195.1±1.0 a
**Sty01-Cter**	41.7±0.6 b	48.6±1.1 a	49.0±0.5 b	61.8±1.4 a	201.2±2.1 a

Different letters indicate significant differences according to Kruskal-Wallis test.
