# Supplementary material for: Stylet cuticular gene-directed mutagenesis impairs the pea aphid vector capacity to transmit a plant virus
Source: PLoS Pathog. 2025 May 23;21(5):e1013192. doi: 10.1371/journal.ppat.1013192 (PMC12140417; doi:10.1371/journal.ppat.1013192)
Supplement: S3 Table — Ac: acrostyle; Ed: edge at the apex of maxillary stylets. The fluorescence was scored “Strong labeling” for a strong signal evenly detected on the acrostyle surface; “Weak labeling” for a weak signal detected as dots or partially distributed on the acrostyle surface. Total over two independent biological replicates. (PDF) [file ppat.1013192.s009.pdf]

S3 Table. Detection of Stylin peptides at the surface of adult maxillary stylets in wild-type and mutant aphid lines. Ac: acrostyle; Ed: edge at the apex of maxillary stylets. The fluorescence was scored "Strong labeling" for a strong signal evenly detected on the acrostyle surface; "Weak labeking" for a weak signal detected as dots or partially distributed on the acrostyle surface. Total over two independent biological replicates.

**Antibody ID**	**Aphid line**	**Target stylin**	**Maxillary stylets observed**					
			Total number	Unlabeled number	Labeled number	Phenotypes of labeled stylets		
						Location	Strong labeling	Weak labeling
Anti-1-11	WT	01/02	44	4	40	Ac	87.5%	12.5%
	Sty01-KO	02	48	6	42	Ac	88.1%	11.9%
	Sty01-Cter	02	46	4	42	Ac	83.3%	16.7%
Anti-1-15	WT	03	51	1	50	Ac	74.0%	26.0%
	Sty01-KO	03	50	4	46	Ac/Ed	10.9%	89.1%
	Sty01-Cter	03	55	0	55	Ac	90.9%	9.1%
Anti-1-16	WT	04/04bis	33	8	25	Ac	0%	100%
	Sty01-KO	04/04bis	30	9	21	Ac	0%	100%
	Sty01-Cter	04/04bis	30	8	22	Ac	0%	100%
