# Supplementary material for: Stylet cuticular gene-directed mutagenesis impairs the pea aphid vector capacity to transmit a plant virus
Source: PLoS Pathog. 2025 May 23;21(5):e1013192. doi: 10.1371/journal.ppat.1013192 (PMC12140417; doi:10.1371/journal.ppat.1013192)
Supplement: S4 Table — (PDF) [file ppat.1013192.s010.pdf]

S4 Table. List of the oligonucleotides used for qRT-PCR analyses

Name	Accession ID	Sequence (5' to 3')	Amplicon size	Reference
**Mdh2-F**[a]<br>**Mhd2-R**	NM_001126203.2	TCCTGTTATCGGCGGACATT<br>ATGCCATAGACAGGGTTGCA	176	N/A
**Ndufs8-F**[b]<br>**Ndufs8-R**	NM_001162323.2	CCTGCAAACTGTGTGAAGCT<br>ACAATGGCATCTACGGGACA	160	N/A
**qSty01-F**[c]<br>**qSty01-R**	NM_001162314.1	ATGCAGGTCACTTTTGCCGTAT<br>GGCTCTGGATTCCGGGTTCA	90	N/A
**qSty02-F**[d]<br>**qSty02-R**	NM_001162671.2	GGTCACATTAGTCCTCTTCATCG<br>TGACTTCTTGTTCTTGGGACAG	107	[15]
**qSty03-F**[e]<br>**qSty03-F**	NM_001161959.2	ATCGTCAGTCACACTCGAGAAG<br>GAGTCGTGGGTTGAAACTGTTG	103	[15]
**qSty04-F**[f]<br>**qSty04-R**	NM_001172260.1/<br>NM_001172268.2	ATCGCCTCCGTCGCCTTATTTC<br>GGGTTTTGCCCGTTGTTGTAG	135/159	N/A

[a] Mitochondrial malate dehydrogenase

[b] NADH dehydrogenase (ubiquinone) Fe-S protein 8

[c] stylin-01

[d] stylin-02

[e] stylin-03

[f] stylin-04/04bis

N/A, not applicable

15. Deshoux M, Masson V, Arafah K, Voisin S, Guschinskaya N, van Munster M, et al. Cuticular Structure Proteomics in the Pea Aphid Acyrthosiphon pisum Reveals New Plant Virus Receptor Candidates at the Tip of Maxillary Stylets. J Proteome Res. 2020/01/29 ed. 2020;19: 1319–1337. doi:10.1021/acs.jproteome.9b00851
