# Supplementary material for: Stylet cuticular gene-directed mutagenesis impairs the pea aphid vector capacity to transmit a plant virus
Source: PLoS Pathog. 2025 May 23;21(5):e1013192. doi: 10.1371/journal.ppat.1013192 (PMC12140417; doi:10.1371/journal.ppat.1013192)
Supplement: S5 Table — Feeding behavior recorded for 10 minutes on turnip plants infected with CaMV (21 dpi) for EPG variables related to virus acquisition, and for 10 minutes on young healthy turnip plants for EPG variables related to virus inoculation. Time is expressed in seconds (mean values ± SE). (PDF) [file ppat.1013192.s011.pdf]

S5 Table. Comparison of the feeding behavior of N2 nymphs of WT and mutant *A. pisum* lines. Feeding behavior recorded for 10 minutes on turnip plants infected with CaMV (21 dpi) for EPG variables related to virus acquisition, and for 10 minutes on young healthy turnip plants for EPG variables related to virus inoculation. Time is expressed in seconds (mean values ± SE).

Variables	Aphid line	VIRUS ACQUISITION			VIRUS INOCULATION		
		**N**	**WDI**	***p***	**N**	**WDI**	***p***
**t > 1pd**	WT	21	6.90±0.50	0.12	22	9.02±1.52	0.56
	Sty01-KO	21	7.13±1.02		21	9.66±1.24	
	Sty01-Cter	21	9.85±2.19		21	9.01±1.03	
**d_1pd**	WT	22	3.67±0.10	0.52	22	3.98±0.20	0.13
	Sty01-KO	21	3.48±0.11		21	3.59±0.12	
	Sty01-Cter	21	3.52±0.11		21	4.09±0.21	
**d_1pd II-1**	WT	22	1.37±0.07	0.60	22	1.56±0.07	0.11
	Sty01-KO	21	1.37±0.06		21	1.33±0.07	
	Sty01-Cter	21	1.31±0.06		21	1.45±0.07	
**d_1pd II-2**	WT	22	1.18±0.06	0.74	22	1.10±0.06	0.22
	Sty01-KO	21	1.11±0.05		21	1.15±0.07	
	Sty01-Cter	21	1.16±0.07		21	1.29±0.08	
**d_1pd II-3**	WT	22	1.12±0.08	0.53	22	1.33±0.14	0.91
	Sty01-KO	21	1.00±0.06		21	1.11±0.08	
	Sty01-Cter	21	1.05±0.07		21	1.35±0.14	

WDI: Waveform duration per insect.

First C to first pd: time from the beginning of the first probe to first pd.

First pd: first intracellular puncture.

II-1, 2, 3: pd subphases indicated here for the first pd.

p-values according to a Kruskal-Wallis test (P≤0.05 would be considered statistically significant).
