# Supplementary material for: Stylet cuticular gene-directed mutagenesis impairs the pea aphid vector capacity to transmit a plant virus
Source: PLoS Pathog. 2025 May 23;21(5):e1013192. doi: 10.1371/journal.ppat.1013192 (PMC12140417; doi:10.1371/journal.ppat.1013192)
Supplement: Attachment — Submitted filename: PPATHOGENS-D-25-00024_reviewer - edit.pdf [file ppat.1013192.s014.pdf]

PLOS Pathogens

Stylet cuticular gene-directed mutagenesis impairs the pea aphid vector capacity to transmit a plant virus

--Manuscript Draft--

**Manuscript Number:**	PPATHOGENS-D-25-00024
**Full Title:**	Stylet cuticular gene-directed mutagenesis impairs the pea aphid vector capacity to transmit a plant virus
**Short Title:**	Aphids' stylet protein disruption impedes virus transmission
**Article Type:**	Research Article
**Section/Category:**	Plant Pathogens
**Keywords:**	CRISPR-Cas9, cuticular protein, hemipteran, vector, virus transmission
**Abstract:**	Aphids are major agricultural pests, notably because they transmit nearly 30% of known plant viruses, including non-circulative ones. They can be collected and dispersed rapidly among crops, while aphids feed on infected plants. Most of these viruses are retained on receptors located on the cuticle of the stylet tip. The acrostyle, a cuticular micro-territory at the apex of aphid stylets, has been identified for its ability to retain the Cauliflower mosaic virus (CaMV). The acrostyle displays cuticular proteins, known as stylins, with exposed domains accessible at the virus-vector interface. RNAi-mediated silencing of Stylin-01 designated this protein as the prime candidate receptor of CaMV. However, the results were incomplete due to the transient effect and highlighted the need for stable mutants to advance our knowledge and validate these putative virus receptors. Here, we characterized the phenotype of two pea aphid Stylin-01 mutant lines, the first generated with CRISPR/Cas9 in this hemipteran. We showed that Stylin-01 mutations significantly disrupt CaMV transmission and impair the acrostyle's ability to bind the CaMV helper protein P2. Stylin-01 mutations also reshape the distribution of other stylins on the surface of mutant aphid stylets. In addition, Stylin-02, the putative ortholog of Stylin-01, is overexpressed in the mutant lines, pointing out a potential partial complementation of Stylin-01 in its structural role but not for virus transmission. In conclusion, this study, using the first stable aphid mutant lines, allows the characterization of the central role of Stylin-01 virus receptor in CaMV transmission.
**Additional Information:**	
**Question**	**Response**
**Government Employee**<br><br>Are you or any of the contributing authors an employee of the United States government?<br><br>Manuscripts authored by one or more US Government employees are not copyrighted, but are licensed under a CC0 Public Domain Dedication, which allows unlimited distribution and reuse of the article for any lawful purpose. This is a legal requirement for US Government employees.<br><br>This will be typeset if the manuscript is	No - No authors are employees of the U.S. government.

Powered by Editorial Manager® and ProduXion Manager® from Aries Systems Corporation

accepted for publication.	
**Financial Disclosure**<br><br>Enter a financial disclosure statement that describes the sources of funding for the work included in this submission. Review the submission guidelines for detailed requirements. View published research articles from *PLOS Pathogens* for specific examples.<br><br>This statement is required for submission and **will appear in the published article** if the submission is accepted. Please make sure it is accurate.<br><br>**Funded studies**<br>Enter a statement with the following details:<br>• Initials of the authors who received each award<br>• Grant numbers awarded to each author<br>• The full name of each funder<br>• URL of each funder website<br>• Did the sponsors or funders play any role in the study design, data collection and analysis, decision to publish, or preparation of the manuscript?<br><br>Did you receive funding for this work?	Yes
Please add funding details.<br>as follow-up to "**Financial Disclosure**<br><br>Enter a financial disclosure statement that describes the sources of funding for the work included in this submission. Review the submission guidelines for detailed requirements. View published research articles from *PLOS Pathogens* for specific examples.<br><br>This statement is required for submission and **will appear in the published article** if the submission is accepted. Please make sure it is accurate.	MU<br>Grants ANR-15-CE20-0011 and ANR-21-CE20-0001<br>French National Research Agency<br>https://anr.fr/en/<br><br>MU<br>GCEag, OPP1130147<br>Bill and Melinda Gates Foundation<br>https://www.gatesfoundation.org/<br><br>YF<br>Grant 202103250004<br>Chinese Scholarship Council<br>https://www.chinesescholarshipcouncil.com/<br><br>The funders had no role in the study design, data collection and analysis, decision to publish, or preparation of the manuscript.

Powered by Editorial Manager® and ProduXion Manager® from Aries Systems Corporation

**Funded studies**<br>Enter a statement with the following details:<br>• Initials of the authors who received each award<br>• Grant numbers awarded to each author<br>• The full name of each funder<br>• URL of each funder website<br>• Did the sponsors or funders play any role in the study design, data collection and analysis, decision to publish, or preparation of the manuscript?<br><br>Did you receive funding for this work?"	
Please select the country of your main research funder (please select carefully as in some cases this is used in fee calculation).<br>as follow-up to "**Financial Disclosure**<br><br>Enter a financial disclosure statement that describes the sources of funding for the work included in this submission. Review the submission guidelines for detailed requirements. View published research articles from *PLOS Pathogens* for specific examples.<br><br>This statement is required for submission and **will appear in the published article** if the submission is accepted. Please make sure it is accurate.<br><br>**Funded studies**<br>Enter a statement with the following details:<br>• Initials of the authors who received each award<br>• Grant numbers awarded to each author<br>• The full name of each funder<br>• URL of each funder website<br>• Did the sponsors or funders play any role in the study design, data collection and analysis, decision to publish, or preparation of the manuscript?<br><br>Did you receive funding for this work?"	FRANCE - FR
**Competing Interests**	The authors have declared that no competing interests exist.

On behalf of all authors, disclose any competing interests that could be perceived to bias this work.<br><br>This statement will be typeset if the manuscript is accepted for publication.<br><br>Review the instructions link below and PLOS Pathogens' competing interests policy to determine what information must be disclosed at submission.	
**Data Availability**<br><br>Provide a **Data Availability Statement** in the box below. This statement should detail where the data used in this submission can be accessed. This statement will be typeset if the manuscript is accepted for publication.<br><br>Before publication, authors are required to make all data underlying their findings fully available, without restriction. Review our PLOS Data Policy page for detailed information on this policy. Instructions for writing your Data Availability statement can be accessed via the Instructions link below.	All relevant data are within the paper and its Supporting Information files.

Powered by Editorial Manager® and ProduXion Manager® from Aries Systems Corporation

Click here to access/download;Manuscript;Fu-et-al_Manuscript.pdf

Stylet cuticular gene-directed mutagenesis impairs the pea aphid vector capacity to transmit a plant virus

Yu Fu[1], Maëlle Deshoux[1], Bastien Cayrol[1], Sophie Le Blaye[1], Emma Achard[1], Sylvie Hudaverdian[2], Romuald Cloteau[2], Elodie Pichon[1], Elian Strozyk[1], Nathalie Prunier-Leterme[2], Emmanuelle Jousselin[3], Nicolas Sauvion[1], Gaël Thébaud[1], Gaël Le Trionnaire[2], Stefano Colella[1*], Marilyne Uzest[1*]

[1] PHIM Plant Health Institute, Univ Montpellier, INRAE, IRD, CIRAD, Institut Agro, 34980 Montpellier, France

[2] IGEPP INRAE, Institut Agro, Univ Rennes, 35653 Le Rheu, France

[3] CBGP INRAE, Univ Montpellier, 34988 Montpellier, France

*Corresponding authors

Email: stefano.colella@inrae.fr (SC); marilyne.uzest@inrae.fr (MU)

Short title: Aphids' stylet protein disruption impedes virus transmission

Abstract

Aphids are major agricultural pests, notably because they transmit nearly 30% of known plant viruses, including non-circulative ones. They can be collected and dispersed rapidly among crops, while aphids feed on infected plants. Most of these viruses are retained on receptors located on the cuticle of the stylet tip. The acrostyle, a cuticular micro-territory at the apex of aphid stylets, has been identified for its ability to retain the Cauliflower mosaic virus (CaMV). The acrostyle displays cuticular proteins, known as stylins, with exposed domains accessible at the virus-vector interface. RNAi-mediated silencing of Stylin-01 designated this protein as the prime candidate receptor of CaMV. However, the results were incomplete due to the transient effect and highlighted the need for stable mutants to advance our knowledge and validate these putative virus receptors. Here, we characterized the phenotype of two pea aphid Stylin-01 mutant lines, the first generated with CRISPR/Cas9 in this hemipteran. We showed that Stylin-01 mutations significantly disrupt CaMV transmission and impair the acrostyle's ability to bind the CaMV helper protein P2. Stylin-01 mutations also reshape the distribution of other stylins on the surface of mutant aphid stylets. In addition, Stylin-02, the putative ortholog of Stylin-01, is overexpressed in the mutant lines, pointing out a potential partial complementation of Stylin-01 in its structural role but not for virus transmission. In conclusion, this study, using the first stable aphid mutant lines, allows the characterization of the central role of Stylin-01 virus receptor in CaMV transmission.

Author Summary

Aphids are severe agricultural pests, well known as major vectors of hundreds of plant viruses. They feed on plant sap using their specialized mouthparts, the stylets. At their tip, they house the acrostyle, a cuticular cell-free microstructure with adhesive properties featuring the stylin proteins that bind aphid effectors, critical molecules for plant-insect interactions. Non-circulative viruses have evolved to bind aphids' stylet tips and stylins to be transported from plant to plant. Understanding stylins' role in virus-vector-plant interactions and identifying virus receptors constitute a stepping stone for developing innovative crop protection approaches. Using the first CRISPR-Cas9-generated aphid mutants, we demonstrate the crucial role of Stylin-01 as a receptor of the plant virus CaMV.

Introduction

Aphids are hemipteran phloem-feeders that are major pests for agriculture. They are found in most cultivated ecosystems, mainly in temperate regions, but not exclusively [1,2]. In addition to the direct damages inflicted on the plants on which they pullulate, they are vectors of a wide variety of plant pathogenic viruses, significantly impacting plant health and crop yields [3,4]. The transmission mechanisms have been extensively studied in aphids, which exhibit the broadest diversity of virus-vector interaction categories among other insects [5]. Aphids are particularly effective vectors of non-circulative viruses comprising hundreds of viral species. These viruses bind on the cuticle of the aphid's mouthparts, either in the foregut or, more commonly, in

maxillary stylets, where they are specifically and transiently retained [6,7]. Unlike circulative viruses, non-circulative ones need not be internalized in their vector for transmission to new plants. Aphids can acquire and inoculate non-circulative viruses within seconds during feeding probes they make to test the suitability of both host and nonhost plants before eventually fleeing to more compatible hosts [8–10]. Controlling viral epidemics in these conditions is challenging. Developing approaches that effectively prevent or inhibit virus transmission by aphids requires an in-depth understanding of virus-vector interactions, including the identification of virus receptors in vectors' mouthparts.

Cauliflower mosaic virus (CaMV) is the virus model for which work on receptor identification is the most advanced. The CaMV is transmitted by aphids using a helper strategy. Its retention in the insect stylets is mediated by a helper component, the non-structural viral protein P2 [11]. Its aphid receptors are located on the surface of the acrostyle, a cuticular micro-territory at the apex of aphid stylets with unique surface properties distinct from the rest of the cuticle [12,13]. This area also likely harbors receptors for numerous other non-circulative viruses [8]. Six cuticular proteins, named stylins, were identified at the surface of the acrostyle [14,15]. Therefore, they are all candidate receptors of plant viruses. Five of them (Stylin-01, 02, 03, 04, and 04bis) belong to the most prominent cuticular protein family, defined by the presence of the conserved Rebers and Riddiford Consensus motif (R&R) [16], known as CPR family. These five stylins contain one of the three known distinct forms of the Consensus, the RR-1 chitin-binding domain. CPR family proteins, particularly RR-1 proteins, have recently emerged as key players in the vector transmission of circulative and non-circulative plant viruses [14]. At the acrostyle surface, stylins also interacts with salivary effectors, suggesting their original role in aphid-plant interactions [17], that viruses have hijacked to ensure their transport from plant to plant.

Stylin-01 and Stylin-02 were the first acrostyle proteins identified [14]. They share a high degree of sequence similarity ~~and~~ possess an almost identical C-terminal domain, detected all over the acrostyle surface~~,~~ where the CaMV helper protein P2 binds [14]. The CaMV receptors are not exposed on the surface of a cell, but ~~they~~ are embedded into an assembly of chitin nanofibres and specific structural proteins making up the cuticle of aphid cell-free stylets. ~~Due to the impossibility of~~ obtaining folded functional stylins outside their natural environment, classic protein-protein interaction approaches to determine which stylin interacts with the CaMV P2 protein are not feasible [14]. The role of Stylin-01 and Stylin-02 in CaMV transmission has been investigated in the green peach aphid *Myzus persicae* using RNAi-mediated knockdown. Despite only partial, transient, and highly variable silencing of Stylin-01, this approach allowed us to confirm this protein as a prime candidate for CaMV receptor, since the aphid's ability to transmit the virus was impacted following siRNA treatment. The same was not true when silencing Stylin-02 [14]. RNAi-mediated gene silencing does not allow formal demonstration of gene function. To overcome this limitation, we ~~decided to take~~ advantage of the CRISPR-Cas9 mutagenesis approach, ~~developing~~ Stylin-01 mutant lines in the pea aphid *Acyrthosiphon pisum*, a vector aphid species able to transmit CaMV [18].

Here, we present a comprehensive characterization of the phenotypic traits of two Stylin-01 edited aphid lines, a complete knock-out, and a mutant that produces a protein modified at its exposed C-terminal domain. Using these unique mutant lines, we demonstrate that a fully functional Stylin-01 is needed for efficient CaMV transmission by aphids. Our work significantly advances the understanding of the role of this cuticular component of the acrostyle, central organ in plant-virus-vector interaction.

Results

Characteristics of the Stylin-01 mutant lines used in this study

To investigate the role of the RR-1 cuticular protein Stylin-01 in CaMV transmission and determine whether disrupting *Stylin-01* gene would lead to a total absence of virus transmission, we used stably edited Stylin-01 *A. pisum* lines generated using CRISPR-Cas9 targeted mutagenesis as described in a previous methodology development paper [18]. We selected three genotypes from the same parental crossing for our experiments: the wild-type (WT) unmodified sister line used as a control and two mutant lines, Sty01-KO and Sty01-Cter (respectively L2 and L14 lines in Le Trionnaire et al. 2019) [18]. In the Sty01-KO line, a deletion in the first allele and multiple re-arrangements in the second one generated frameshift mutations, changes in the amino acids (AA) sequences, and early translational stop signals. This resulted in truncated alleles and total loss of function (Fig 1A and S1 Fig). In the Sty01-Cter line, allele 1 encodes a shorter protein lacking the 11 C-terminal AA that form part of the acrostyle surface-exposed domain susceptible to interacting with viruses, and allele 2 encodes a protein in which the last 11 AA have been replaced by an entirely new sequence of 12 AA not present in the wild-type protein (Fig 1A and S1 Fig). The 3D structures of the Sty01-Cter mutated Stylin-01 proteins predicted by AlphaFold2 closely resemble the one of Stylin-01 wild-type, with a C-terminal domain partly folded in the alpha helix (in red, Fig 1A). The Sty01-KO mutant aphids no longer produce Stylin-01 protein and synthesize a stylet bundle devoid of this RR-1 cuticular protein (Fig1B, S2 Fig, and S1 Table). In contrast, the mutated Stylin-01 protein is produced in Sty01-Cter aphids, and the isoform encoded by allele 2 is detected in whole-body crude extracts (Fig. 1B) and is detected at the apex of maxillary stylets

by immunofluorescence microscopy (Fig. 1C). The three aphid lines have a similar developmental time (for details, see Supporting Information, S3 Fig and S2 Table).

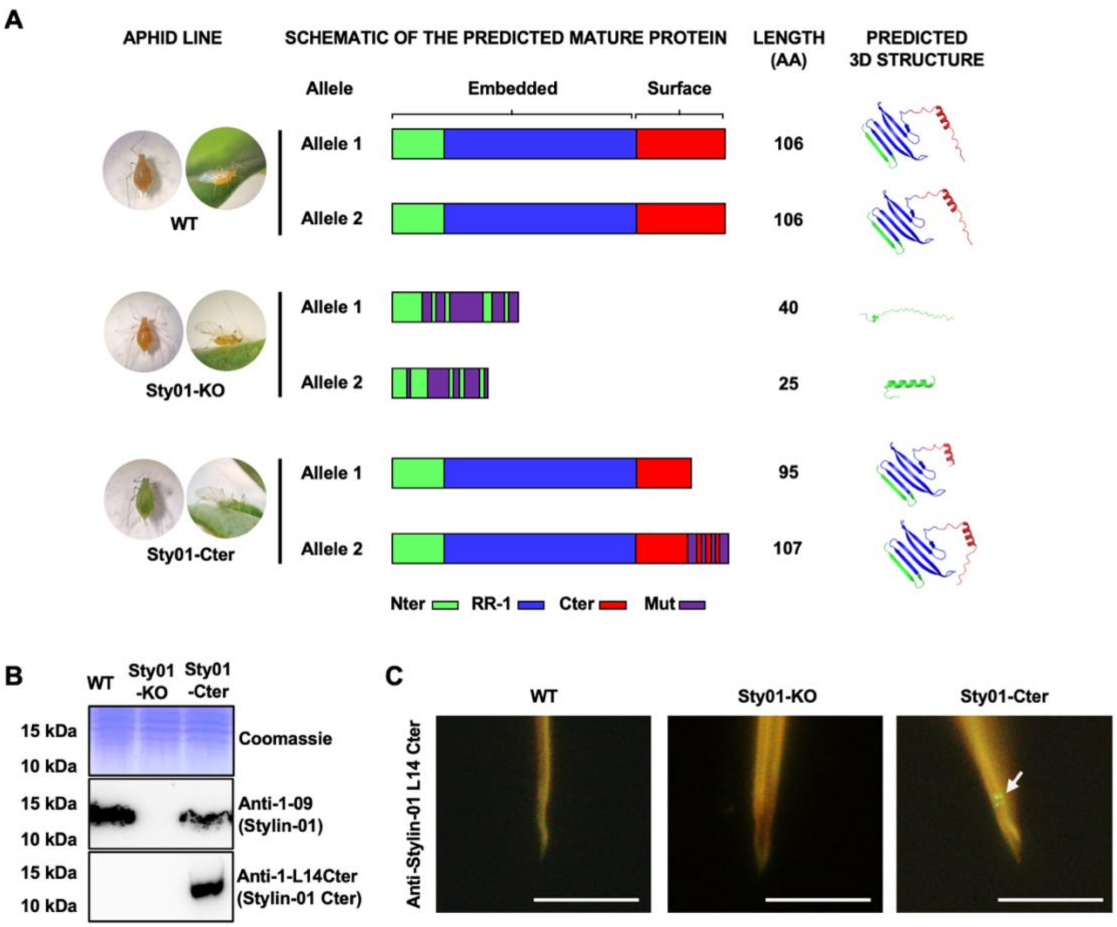

Fig 1. CRISPR-Cas 9 stylin-01 edited aphid lines (Sty01-KO and Sty01-Cter) and wild-type non edited line (WT). (A) Schematic representation of the domains of the mature proteins and their respective 3D structure predicted by AlphaFold2. The N-terminal (Nter), C-terminal (Cter) and RR-1 chitin-binding domains are indicated in green, red and blue respectively. Mutations in the amino acid sequences (Mut) are indicated in purple. (B) Detection of Stylin-01 proteins in *A. pisum* lines by western blot analysis. Whole-body crude extract proteins from WT, Sty01-KO, and Sty01-Cter aphids (lanes 1, 2 and 3, respectively) were analyzed by SDS-PAGE and stained with Coomassie blue to compare the protein amount loaded on the gel, or transferred onto a nitrocellulose membrane prior to immunolabeling with anti-1-09 or anti-1-L14Cter antibodies (S2A and B, S1 Table). (C) Detection of Stylin-01 Cter mutant protein in dissected stylets of the 3 aphid lines using immunofluorescence labeling and confocal microscopy. The mutant protein is detected as green

dots at the tip of maxillary stylets of Sty01-Cter aphids (indicated with a white arrow). Stylets of the wild-type or Sty01-KO aphids are not labeled by this antibody. Bars: 10 µm.

Disruption of Stylin-01 impairs the capacity of aphids to transmit the cauliflower mosaic virus

We investigated the impact of Stylin-01 mutations on CaMV transmission efficiency by conducting transmission test experiments with cohorts of wild-type and Stylin-01 mutant aphids. Our results indicate that the transmission of CaMV was not completely abolished, regardless of the mutations considered. However, both mutants were impaired in their capacity to transmit CaMV compared to control aphids (Fig 2). The lowest transmission efficiency was obtained for the Sty01-KO mutant with a mean of 3.8±0.8% transmission efficiency, representing a highly significant difference of 84% compared to the transmission efficiency of the wild-type TNJ4 (23.6±1.7%, $P<0.001$). Sty01-Cter aphids also showed an intermediate transmission efficiency lower than WT (13.5±2.5% transmission, $P=0.011$) but higher than Sty01-KO ($P=0.001$).

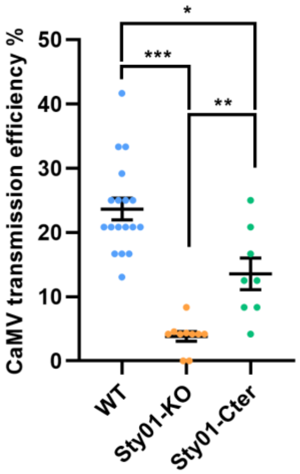

Fig 2. Impact of Stylin-01 mutations on CaMV transmission efficiency. The values are presented as the means ± SE of all independent replicates. Asterisks indicate significant differences according to binomial generalized linear model followed by a post-hoc Tukey's honestly significant difference (HSD) test (GLM, $P < 0.05$).

Mutations compromise the acrostyle's ability to bind the CaMV P2 helper protein

We tested whether the decrease in transmission efficiency reflected a reduced ability of the mutated aphid stylets and acrostyle to interact with the CaMV P2 helper protein. We therefore incubated dissected stylets of WT, Sty01-KO, and Sty01-Cter aphids with the viral protein fused to GFP (P2-GFP). Maxillary stylets were grouped into 3 classes (positive, partial, negative) according to the intensity and location of the observed labeling (Fig. 3A, see Materials and Methods for details). The results show a significant reduction in the number of positively labeled stylets for both mutant lines compared to WT aphids (Fig. 3B). In contrast with the maxillary stylets in WT that were all able to bind P2-GFP strongly along the acrostyle surface, 23.3% and 19.8% of the Sty01-KO and Sty01-Cter mutants' stylets respectively showed a statistically significant P2 binding defect. Consistent with the virus transmission defect, our results indicate that the stylets of mutant aphids have altered binding capacities for CaMV P2.

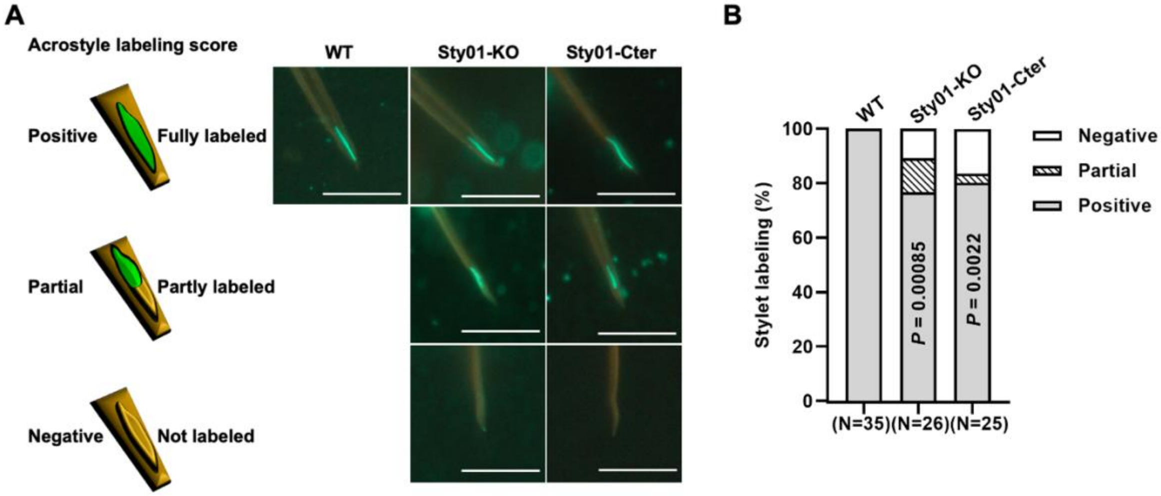

Fig 3. *In vitro* interaction assays between dissected aphid stylets and the viral protein CaMV P2 fused to GFP (P2-GFP). (A) Representative images of the detection of P2-GFP on the acrostyle of dissected stylets of WT, Sty01-KO and Sty01-Cter adults. Stylets were scored (positive, partial, negative) according to the detection and localization of the fluorescence labeling as shown in the schematic diagram on the left panel. Scale bars represent 10 µm. (B) Proportion of maxillary stylets labeled for each category out of the total number of maxillary stylets observed. The number of positive stylets was significantly lower in the two mutant lines Sty01-KO and Sty01-Cter than in the wild-type aphid line (P-values from a binomial GLM), "N" indicates the total number of maxillary stylets observed for each treatment from three (Sty01-KO, Sty01-Cter) or four (WT) biological replicates.

No ultrastructural changes in the acrostyle of mutant aphids

To examine whether the mutations ~~introduced~~ affect the ultrastructure of aphid stylets, particularly the apical part of maxillary stylets housing the acrostyle, we conducted a series of observations of the stylet tip using transmission electron microscopy (TEM). The TEM approach allows the observation of the acrostyle as a thin, distinguishable electron-dense area in the common food/salivary canal of *A. pisum* and other aphid species on stylet tip cross-sections [12,13]. As shown in the images (Fig 4), the maxillary stylets of both mutants and WT exhibit a

clear layer surrounding the stylet cuticle, which becomes a darker line in the bottom bed of the stylet, typical of the acrostyle. Therefore, TEM could not highlight any detectable difference in this area at this scale.

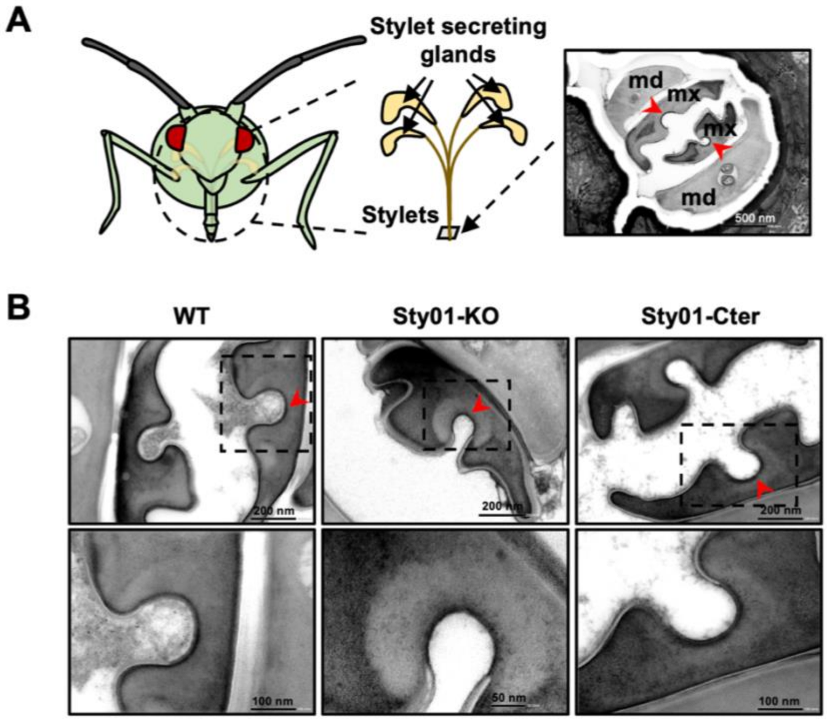

Fig 4. Observation of the stylet ultrastructure in wild-type and Stylin-01 mutant aphids using transmission electron microscopy (TEM). (A) Schematic representation of the approximative position of the cross sections of the apical part of aphid stylets observed under TEM in (B). The stylet bundle visible on the electron micrograph shows the two mandibular stylets (md) surrounding the two maxillary stylets (mx). (B) Electron micrographs of cross sections of the maxillary stylets of each aphid line (upper panel), observed at higher magnification in the bottom panel. The acrostyle, visible as an electron-dense area in the maxillary stylets, is indicated with a red arrowhead in (A) and (B, upper panel). Bars: 500 nm in (A), 200 nm in (B, upper panel), 50-100 nm in (B, bottom panel).

Modification of stylins' distribution at the surface of stylets of mutant aphids

Using a set of well-defined specific antibodies (S2 Fig and S1 Table), we further investigated the distribution of RR-1 stylins (Stylin-01, 02, 03, 04, and 04bis) in the maxillary stylets of the wild-type and ~~the~~ mutant lines using immunofluorescence microscopy. The anti-1-11 antibody directed against the 15 C-terminal AAs of Stylin-01 and Stylin-02 can detect both proteins (S2A and 2C Fig). Our results show that, in both mutant aphid lines, stylet labeling with anti-1-11 antibody is similar to that observed in wild-type aphids (Fig 5), indicating that in the absence of Stylin-01, the C-terminal domain of Stylin-02 is highly accessible all along the acrostyle surface of their stylets. The anti-1-15 antibody targeting Stylin-03 protein reveals that the distribution differs in the stylets of the three aphid lines. Stylin-03 protein is mainly detected as a labeled broken line at the surface of the acrostyle in 74% of the WT and 91% of the Sty01-Cter stylets. On the other hand, in the Sty01-KO mutant Stylin-03 is weakly detected and located at the edges of the apical part of maxillary stylets (Fig 5, S3 Table).

The N-terminal domain of Stylin-04 and 04bis is similarly detected at the apex of maxillary stylets of wild-type and mutants, but with a weaker signal on the acrostyle edge and in its upper part in the vicinity of the food canal (Fig 5).

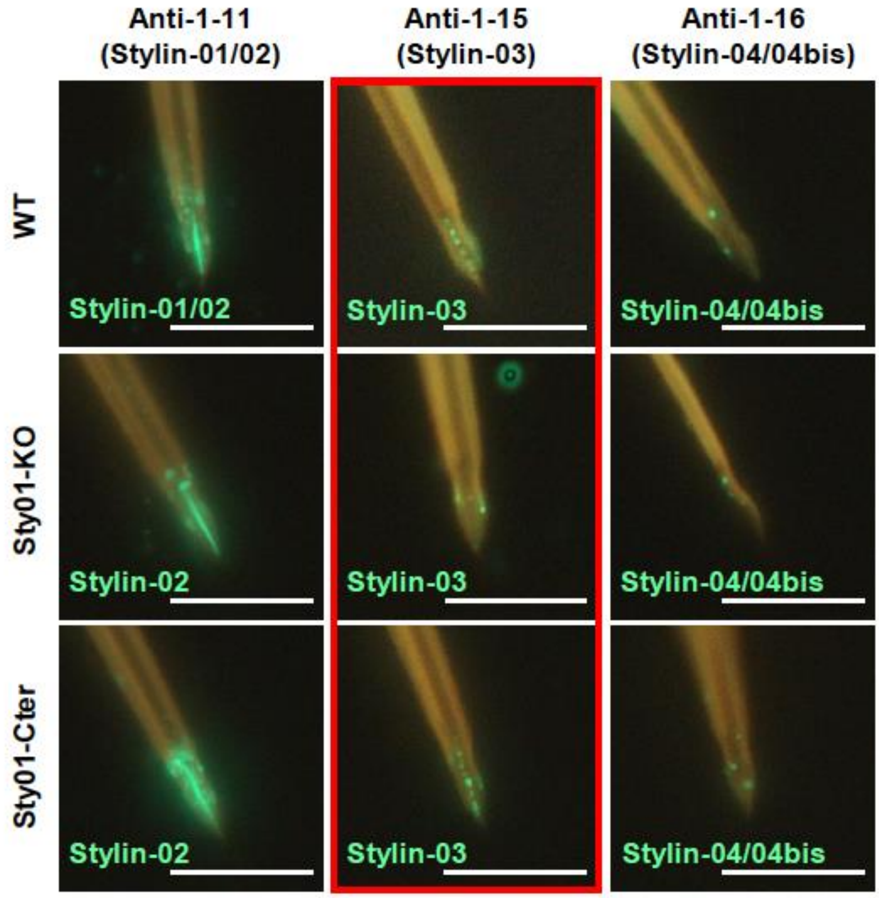

Fig 5. Distribution of RR-1 stylins in wild-type and Stylin-01 mutant aphids. Representative images of labeling of stylin peptides observed in maxillary stylets of WT, Sty01-KO and Sty01-Cter aphids. The antibodies used and the stylins they theoretically target is indicated above the images (S2 Fig and S1 Table). The stylins detected in aphid stylets are indicated in green at the bottom of each image. The main difference observed at the acrostyle surface is underlined in red. Bars: 10 µm.

Loss of Stylin-01 is associated with the upregulation of Stylin-02 during stylet synthesis.

To evaluate whether the introduced mutations had an impact on the expression of other RR-1 Stylins of the acrostyle, we quantified their expression at the time of adult stylet synthesis based on our characterization of the stylet development (i.e., towards the end of the last nymphal stage N4) [19]. As the developmental time of the aphid nymphs was almost identical for all the lines (S2 Table and S3 Fig), we sampled all N4 nymphs at the time of peak Stylin expression 46 h post-molt as precisely determined in a previous study [20]. Thus, synchronized WT, Sty01-KO, and Sty01-Cter aphids were followed individually from the end of N3 stage to the time of sampling. We performed RT-qPCR analyses using specific primers for *stylin-01*, *-02*, and *-03*, and primers amplifying indiscriminately the highly homologous *stylin-04* and *stylin-04bis* transcripts (S4 Table). The expression patterns of *rr1 stylins* genes in WT and Sty01-Cter lines were similar, with *stylin-01* being the most highly expressed. A residual expression of *stylin-01* gene is detected in the Sty01-KO line as expected, as the PCR primers for *stylin-01* target a region upstream of the mutations in the two alleles. Stylin-02, on the other hand, was the most highly expressed stylin in the Sty01-KO mutant (Fig 6). Consistently, more Stylin-02 protein was detected in whole-body crude extracts of mutant aphids than wild-type aphids (S4 Fig). In contrast, the expression of *stylin-03, stylin-04* and *04bis* transcripts appeared to be unaffected in Sty01-KO and Sty01-Cter mutants (Fig 6). Our results indicate that *stylin-02* expression is upregulated in Sty01-KO nymphs N4 during adult stylet synthesis.

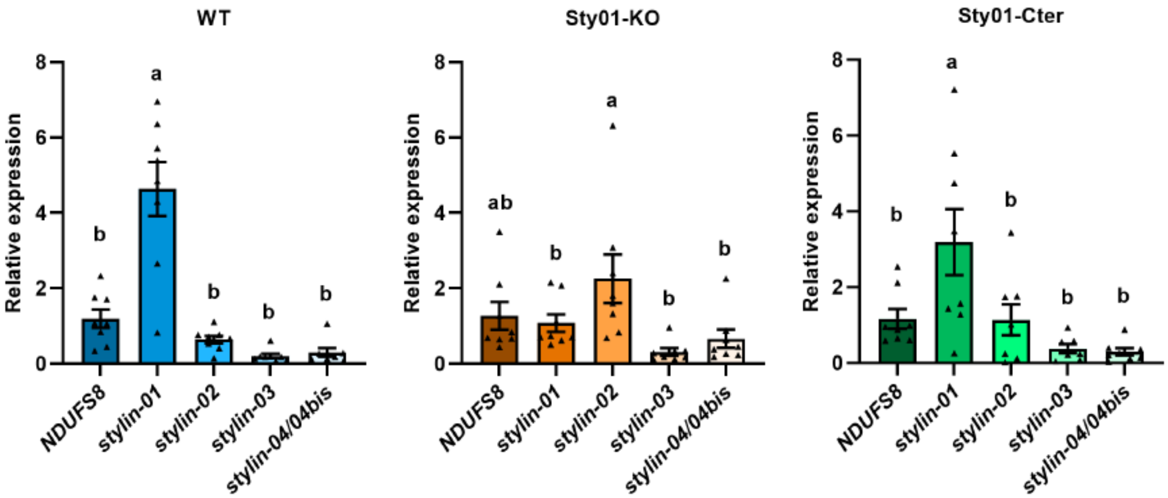

Fig 6. *Stylin-02* is upregulated in the Sty01-KO mutant during the synthesis of adult stylets. Relative expression levels of stylins at N4 instar 46 h post-molt were measured in WT and mutant lines. The gene encoding NADH dehydrogenase [ubiquinone] iron-sulfur protein 8 (NDUFS88) was used as internal control to compare the expression of all RR-1 stylins in each aphid line. The values are presented as the means ± SE (n = 7-8 biological replicates from two independent experiments, different letters indicate significant differences according to one-way ANOVA followed by the least significant difference (LSD) test.

Disruption of Stylin-01 has no impact on the feeding behavior related to CaMV acquisition and inoculation.

To determine if changes in the acrostyle protein surface of pea aphid mutants affect their feeding behavior during CaMV acquisition and inoculation, and subsequently virus transmission, we monitored the feeding behavior of cohorts of both wild-type and mutant nymphs by electropenetrography (EPG). CaMV can be acquired during brief (i.e., a few seconds) intracellular punctures in the epiderm or mesophyll of infected plants [21] and inoculated within seconds when

the aphid feeds on another plant [22]. We therefore used short recording times compatible with non-circulative transmission [23].

We first compared more precisely the variables related to CaMV acquisition: the time taken for the stylet, from start of the first probe (waveform C), to reach the first punctured cell (waveform pd) in mesophyll tissue (t>1pd) [24], and the duration of subphase II-3 of the first intracellular puncture in mesophyll tissue (d_1pdII-3) corresponding to ingestion of the cell contents and virus uptake [8] (Fig 7A). We observed that most of the nymphs had achieved a first intracellular puncture within 20 seconds after the start of the first probe, whatever the aphid line. The duration of the feeding behavior sub-phase II-3 showed a similar distribution pattern in all individuals of the three *A. pisum* lines, (Fig 7B and S5 Table).

We then focused on EPG variables related to virus inoculation into young turnip plants (Fig 7C). We observed no significant difference in feeding behavior among the three aphid lines during the intercellular path to the first punctured cell and in the time spent in this first cell. In particular, the duration of subphase II-2, previously shown to be associated with CaMV inoculation into plant cells [9] were similar (Fig 7D and S5 Table).

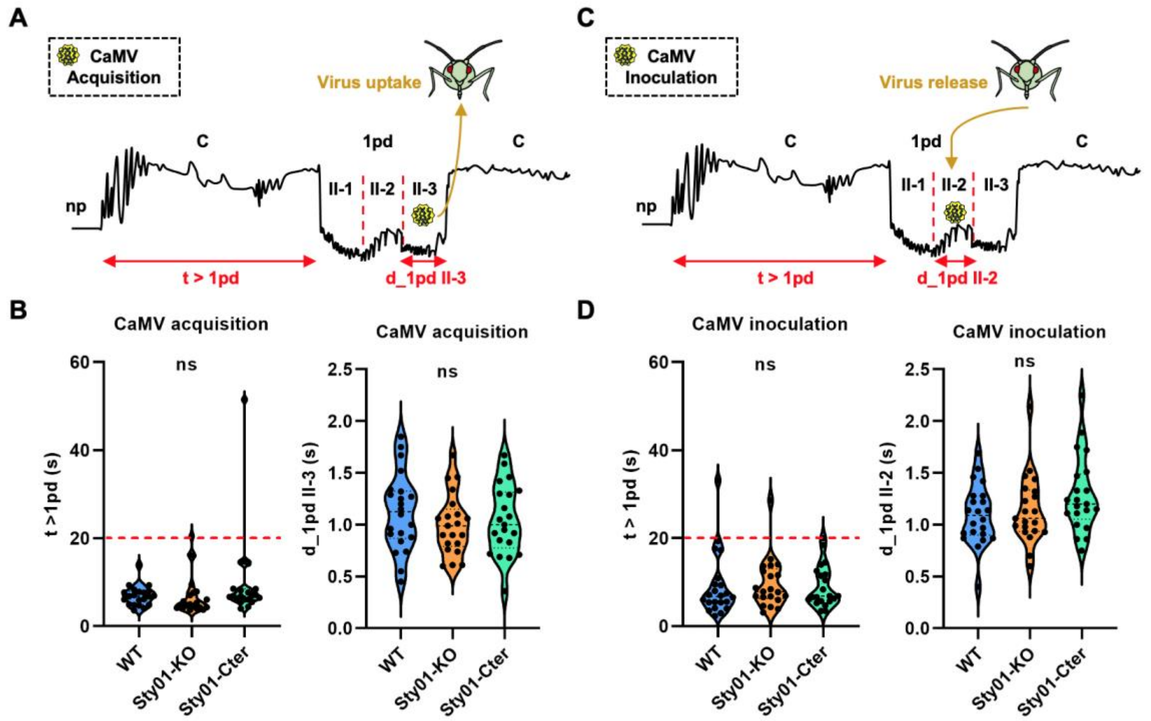

Fig 7. Feeding behavior of wild-type (WT) and mutant *A. pisum* clones related to CaMV acquisition and inoculation. (A, C) Schematic diagrams illustrating the waveforms related to CaMV acquisition and inoculation, respectively. The duration of first C to first pd (time from the beginning of the first probe to the first intracellular puncture) during CaMV acquisition (B) and inoculation (D) show no difference between all aphid clones (n = 21-22, Kruskal-Wallis test, ns = not significant at the 5% level). Below the dotted line are most of the aphids that completed the sequence from the first C to the first pd. The duration of the sub-phase II-3 in the first pd related to ingestion of cell content (B) or the duration of subphase II-2 in the first pd related to CaMV inoculation (D) are similar in all aphid clones (n = 21-22, Kruskal-Wallis test, ns = not significant at the 5% level).

Phylogenetic analyses of RR-1 stylins

Alignment of Stylin-01 (NP_001155786.1) and Stylin-02 (NP_001156143.1) pea aphid proteins showed a high degree of similarity between [14]. In 11 Aphididae species available in GenBank, we recovered putative ortholog sequences for the five RR-1 known stylin genes of the pea aphid

[15]. Using Orthofinder [25], these sequences were assigned to three ortho-groups, one containing Stylin-01 and 02, one for Stylin-03, and one for Stylin-04 and 04bis. Phylogenetic analyses of the Stylin-01 and 02 othogroup separated the ~~recovered~~ proteins into two branches, one for each stylin (S6 Table and S5A Fig). In each branch, the protein tree more or less parallels the more recently established Aphididae species phylogenetic history (S5B Fig) [26,27]. The only incongruence is the relative position of the two species outside the Aphidinae subfamily (i.e. *Sipha flava* and *Cinara cedri*), which varies in each subtree.

Discussion

RR-1 cuticular proteins are major components of the acrostyle surface at the apex of aphid stylets, playing a crucial functional role [15]. In this cell-free cuticular micro-territory RR-1 cuticular proteins confer the ability to transiently bind endogenous and exogenous molecules, such as insect salivary effectors and viruses, respectively. Non-circulative viruses have evolved to interact specifically with the motifs exposed at the surface of the acrostyle, thereby ensuring their transport between plants [14,17]. Receptors of viruses are prime targets for innovative control methods, but their identification in insect vectors remains a considerable technical challenge to overcome, especially for those embedded in the cuticle of the mouthparts. In the absence of standard experimental approaches to characterize virus-receptor interactions, including both *in vitro* and *in vivo* heterologous systems, the role of some RR-1 candidate receptors in virus transmission could until now, be assessed only by RNAi mediated gene expression silencing [14].

In the present study, we used CRISPR-Cas9 edited Stylin-01 stable mutant lines in *A. pisum* [18] to advance our knowledge of the role of Stylin-01 as a receptor of CaMV. The mutations

introduced in the *A. pisum* genome had no apparent impact on the mutant aphids' developmental time and viability, as colonies could be maintained clonally for years under laboratory conditions. The availability of the complete knock-out and a mutant for the C-terminal exposed domain of Stylin-01 allowed us to study its role in plant virus transmission. As expected, the Sty01-KO line exhibits a more ~~severe~~ phenotype than the Sty01-Cter mutant, thus allowing a more comprehensive characterization of the Stylin-01 function.

The ability to transmit the CaMV is almost completely lost in the Sty01-KO mutant line, and highly reduced in the Sty01-Cter compared to the wild-type (Fig 2). These findings are consistent with Webster *et al.* [14], who demonstrated that decreasing significantly *stylin-01* expression in the green peach aphid *Myzus persicae* via RNAi led to a reduction in CaMV transmission. Under the same experimental design with the pea aphid Sty01-KO line, we observe a ~~decrease of~~ 84% in the transmission, which is undoubtedly linked to the complete absence of the Stylin-01 protein. The intermediate phenotype of the Sty01-Cter mutant in CaMV transmission indicates a role of the Stylin-01 C-terminal helix in the binding of the virus to the acrostyle. The mutant lines used in the present study allowed us to perform several complementary experiments that ~~could~~ not ~~have been~~ possible using RNAi silencing, broadening our understanding of the key role of Stylin-01 in CaMV transmission.

The feeding behavior of the mutant aphids is comparable with that of the wild-type during short-term observations with EPG experiments (Fig 7A-D). Notably, all aphids could perform intracellular punctures within 20 seconds after initiating a probe in CaMV-infected and healthy plants, indicating their ability to potentially acquire and inoculate the virus. These data suggest that the observed transmission defect is not due to a change in the insect feeding behavior.

We show here that the viral protein P2 can still bind to the maxillary stylets of both mutant aphids even if with less efficiency (Fig 3), indicating that Stylin-01 is not the sole acrostyle protein with CaMV-binding activities. Disrupting *stylin-01* does not change the overall stylet ultrastructure (Fig 4). However, our immunolabeling data indicate that the distribution of the other RR-1 stylins at the acrostyle's surface is differently affected in the absence of a fully functional Stylin-01. The localization of Stylin-02 on the surface of the Sty01-KO mutant stylets is compatible with the localization of CaMV P2 binding sites, while that of Stylin-03 or Stylin-04/04bis is not (Fig 5). The high similarity between Stylins-01 and -02 in the surface-exposed domain prevents the development of antibodies to detect each one individually. The antibody targeting both putative orthologs showed no difference in the acrostyle labeling between the two mutants and the wild-type lines (Fig 5). We could have expected a difference in the labeling of mutant lines, particularly in the Sty01-KO one where only Stylin-02 is present. Observing a fully labeled acrostyle, in this mutant, leads us to hypothesize that Stylin-02 may replace the missing Stylin-01 protein to organize this cuticular region of the stylet essential for plant-aphid interactions and virus transmission. The hypothesis of the importance of Stylin-02 in the acrostyle structure is also supported indirectly by the observed overexpression of this gene during stylet biogenesis in the mutant lines as detected by quantitative RT-PCR (Fig 6). Furthermore, the evolutionary analyses suggesting that Stylin-01 and Stylin-02 are orthologs supports further our complementation hypothesis. Nonetheless, the distribution of Stylin-02 that is aligned with P2 binding sites does not seem sufficient to complement Stylin-01 function in CaMV transmission.

To date, this study is the first example of the functional demonstration of the role of a gene in a key life history trait of aphids using genome editing. Our work on CRISPR-Cas9 Stylin-01 mutants shows the predominant role of this acrostyle RR-1 cuticular protein in CaMV transmission by the

pea aphid. At the same time, the observed residual, ~~albeit very low,~~ CaMV transmission in the Sty01-KO line shows that Stylin-01 is not the ~~unique and~~ exclusive receptor for this virus. The role of stylins in virus transmission calls for further studies that would benefit from stable mutant lines that ~~empower~~ detailed phenotype characterization and gene function validation.

Materials and Methods

Aphid lines, virus strain, and plants

The three sister lines of *Acyrthosiphon pisum* used in this study, the wild type line (WT) and the two *stylin-01* mutant lines Sty01-KO (full gene knock-out) and Sty01-Cter (Cter deletion) were ~~initially~~ generated in a previous study [18]. ~~They~~ were reared in controlled conditions at 18 °C and a photoperiod of 16/8 h (day/night) on faba bean (*Vicia faba* cv. "Sutton"). The *Cauliflower mosaic virus,* CaMV (isolate Cabb B-JI), was mechanically passaged on turnips (*Brassica rapa* cv. "Just Right"). Healthy and infected plants were maintained at 23/18 °C and a photoperiod of 16/8 h (day/night).

Aphid lines developmental time assessment

To monitor the duration of nymph development, apterous adult aphids were placed on two-week-old faba beans. Experiments were conducted at 18 °C with a photoperiod of 16/8 h (day/night). Adults were allowed to produce one progeny, then eliminated. For each lineage (WT, Sty01-KO, Sty01-Cter), at least 10 nymphs were individually surveyed, ~~and~~ the experiment was repeated twice. The insects were observed every 6 hours, and the duration of each instar was recorded as soon as the nymphs ~~had~~ molted (S2 Table).

Protein extraction and western blot analysis

For each *A. pisum* line, whole-body proteins were extracted from 10 adults in 80 µL phosphate-buffered saline [4.3 mM Na_2HPO_4, 1.4 mM KH_2PO_4, 137 mM NaCl, 2.7 mM KCl, pH 7.3] Samples were separated under denaturing conditions in 15% SDS-PAGE. Gels were stained with Coomassie blue or were transferred onto nitrocellulose membranes (Amersham, Marlborough, USA) using Semi-Dry Blotter. After blocking in Tris-buffered saline (50 mM Tris and 200 mM NaCl, pH 7.4) supplemented with 0.05% Tween 20 (TBST buffer) and 5% (w/v) skimmed milk, membranes were incubated overnight at 4°C with with anti-stylin primary antibodies diluted 1:1000. Membranes were finally incubated for 1 h at room temperature with goat-anti-rabbit IgG-HRP secondary antibodies (sc-2030; Santa Cruz Biotechnology, Dallas, TX), blots were visualized with the G-BOX (Syngene, USA) diluted 1:1500. Interactions were revealed using chemiluminescence according to the manufacturer's instructions (ThermoFisher Scientific).

Aphid transmission assays

Two-day-old aphid nymphs (N2) were collected in glass tubes for 1 h starvation. They were allowed a 2-minute acquisition access period (AAP) on leaf L8 of a CaMV-infected turnip used as a virus source plant 21 days post-inoculation. The nymphs were immediately collected in a glass Petri dish and transferred to 8-day-old turnip test plants for a 3 h inoculation access period (IAP) followed by insecticide treatment. The following design was used in all experiments: 2 nymphs/test plant; 24 test plants/source plant. The aphid transmission efficiency was calculated by recording 28 days post-transmission assay the number of plants presenting viral symptoms out of the total number of test plants used. At least six independent biological replicates were performed for all aphid lines.

In vitro interaction assays on dissected stylets

For immunolabeling, dissected adult stylets were immunolabeled according to Webster and colleagues using primary antibodies at a 1:200 dilution (S1 Table) and secondary Alexa fluor-conjugated antibodies at a 1:400 dilution [28]. Stylets were mounted in Mowiol mounting medium on a glass slide. Images were taken with a confocal laser scanning microscope (ZEISS LSM900). For *in vitro* binding assays with the viral protein P2 fused to GFP (P2-GFP) produced in Sf9 cells, stylets from adult aphids were dissected and incubated with the protein as previously described [12]. Maxillary stylets were scored according to the intensity and location of labeling. They were considered "Positive" if they were strongly labeled on the entire surface of the acrostyle, "Partial" if the labeling was only present on part of the acrostyle, or "Negative" if no labeling was detected. The images were noted by two independent observers. For both types of interaction assays, batches of 8-16 maxillary stylets were observed for each aphid line and experimental condition. Three independent biological replicates were performed.

Sample preparation for transmission electron microscope (TEM)

Acyrthosiphon pisum heads were severed from cold anesthetized adults and fixed in fixative buffer (FB: 0.1 M cacodylate buffer, pH 7.2) containing 4% glutaraldehyde overnight at 4°C. After rinses in FB followed by post-fixation in FB containing 1% osmic acid for 2h at 4°C, samples were dehydrated in a graded acetone series. Finally, they were embedded in Epoxy resin (EmBed 812) using an automated microwave tissue processor for electron microscopy, Leica EM AMW. Sections 60 nm thick were observed in a JEOL JEM 1400 microscope.

Reverse-transcription quantitative PCR

Stylin expression levels were measured in each aphid line at the pre-imaginal stage during the synthesis of adult stylets. N4 nymphs were collected at 46 h post molting, the exact time corresponding to the peak of expression of *stylin 01* and *stylin 02* genes in aphid heads [20]. To that end, wingless adults were put on young faba beans plants and allowed to produce nymphs for 2 h. Cohorts of synchronized aphids were individually followed. N3 nymphs were isolated and screened regularly for N4 emergence. Nymphs were collected 46 h after the beginning of the N4 stage, and their head were immediately dissected. Pools of three heads without antennas were placed in an RNAlater® solution (ThermoFisher Scientific, Waltham, MA, USA) and flash-frozen in liquid nitrogen. Samples were kept at -80 °C until use. Four pools of three heads were collected for each aphid line, and the whole process was repeated twice, resulting in a total of 8 pools of 3 aphid heads per line.

Total RNA was extracted using a RNeasy mini kit (Qiagen, Hilden Germany) and treated with RQ1 RNAse-free DNAse I (Promega Corporation, Madison, WI, USA). First-strand cDNA was synthesized from 200 ng total RNAs using Moloney murine leukemia virus (MMLV) reverse transcriptase (Promega Corporation, Madison, WI, USA) and oligo (dT) as a primer according to the manufacturer's instructions. All quantitative PCRs were performed in triplicates on a LightCycler 480 instrument using a LightCycler 480 SYBR green I master mix (Roche, Penzberg, Germany) according to the manufacturer's recommendations with gene-specific primers (*SI Appendix*, Table S4). Primers for *stylin-01* were designed in the non-mutated 5' end sequence, which is still present in the genomes of both mutant lines. Amplification efficiencies were analyzed with the LinRegPCR 512 software [29]. The genes encoding the mitochondrial malate

dehydrogenase (Mdh2) and the NADH dehydrogenase [ubiquinone] iron-sulfur protein 8 (Ndufs8) exhibit stable expression across samples and tissues. Mdh2 was used as reference gene. Ndufs8 was used as internal control to compare the expression of different stylins within a single aphid line. Relative expressions were determined by using the $2^{-\Delta\Delta CT}$ method [30].

Analysis of aphid feeding behavior

The Electrical Penetration Graph (EPG) method was used as described by [31] to investigate if specific stylet penetration activities known to be associated with acquisition and inoculation were altered in individuals of mutant lines. A Giga-8 DC-EPG device (EPG Systems, Wageningen, The Netherlands) was used to monitor probing and ingestion activities of N2 instar nymphs (for detailed description, see Supporting Information). A total of 21 to 22 aphid nymphs per line were recorded, after they underwent a one-hour pre-acquisition starvation period before EPG recordings began on 21 dpi CaMV-infected plants. To assess potential mutation effects on feeding behavior during acquisition, recordings were conducted for 10 minutes from the start until interruption on CaMV-infected turnip plants. After this 'acquisition phase', the nymphs were individually transferred to young, healthy turnip plants for an additional 10 minutes of recording, constituting the 'inoculation phase'. Each recording was then semi-automaticaly labelled using the software A2-EPG [32], i.e. each recording was scanned by the software but then reviewed manually to check/correct/add labels. The following waveforms in particular were focused: waveforms C (complex of different patterns, correlated with stylet tips somewhere between the epidermis and phloem), potential drop (pd) waveforms (intracellular punctures of plant cells by the stylet tips), subphases II-3 and II-2 of the waveform pd associated with CaMV acquisition and inoculation, respectively [9]. We then calculated the following non-sequential variables for each

individual: time from start of the probe to first pd (t>1pd); duration of pd subphase II-2 (d_pdII-2) ; duration of pd subphase II-3 (d_pdII-3) [24]. From these individual values, we estimated the Waveform Duration per Insect [WDI, as defined by [33]] for each of the three aphid lines.

Protein bioinformatics analysis

The three-dimensional protein structures were predicted via ColabFold v1.5.5 (AlphaFold2 using MMseqs2) [34]. We used an *ad hoc* pipeline to identify, in the Aphididae fully annotated genomes, available in GeneBank (NCBI) [35], putative orthologues of the five stylin genes of *A. pisum*: *Stylin-01* (NM_001162314.1), *Stylin-02* (NM_001162671.2), *Stylin-03* (NM_001161959.2), *Stylin-04* (NM_001172260.1) and *Stylin-04bis* (NM_001172268.2). The protein sequences were used in a BLAST+ [36] search for sequences on GeneBank; the proteins retrieved were analyzed with Orthofinder [25] that returned for the sequences obtained from the 11 aphid species (see *SI Appendix*, Table S6 for full list) three ortho-groups. The protein sequences in the ortho-group of Stylin-01 and 02 were aligned with MAFFT [37,38], and the phylogenetic inference was realized using IQTree2 [39] with 1000 bootstraps and visualized using FigTree [http://tree.bio.ed.ac.uk/software/figtree/].

Statistical analysis

All the data were analyzed using SPSS Statistics 20.0 software or R Stats Package [40], and plotted using GraphPad Prism version 9. Differences in aphid transmission efficiencies were tested using a binomial generalized linear model (GLM) followed by a post-hoc Tukey's honestly significant difference (HSD) test. Differences in stylets labeling were analyzed by GLM. The qPCR data were analyzed using a one-way analysis of variance (ANOVA), followed by the least significant difference (LSD) test. Differences in EPG variables and the duration of nymph development were

analyzed using Kruskal-Wallis test. Different letters indicate significant differences among groups, and asterisks denoted statistical significance between two groups (*P < 0.05, **P < 0.01, and ***P < 0.001).

Acknowledgments

Yu Fu acknowledges the Chinese Scholarship Council (CSC Grant No. 202103250004) and the French National Institute Research for Agriculture, Food and Environment INRAE, for financial support of her PhD studies.

References

1. Emden HFV, Harrington R, editors. Aphids as crop pests. 2nd ed. UK: CABI; 2017. doi:10.1079/9781780647098.0000

2. Simon J-C, Mahéo F, Mieuzet L, Buchard C, Gauthier J-P, Maurice D, et al. Life on the Edge: Ecological Genetics of a High Arctic Insect Species and Its Circumpolar Counterpart. Insects. 2019;10: 427. doi:10.3390/insects10120427

3. Tatineni S, Hein GL. Plant Viruses of Agricultural Importance: Current and Future Perspectives of Virus Disease Management Strategies. Phytopathology®. 2023;113: 117–141. doi:10.1094/PHYTO-05-22-0167-RVW

4. Peters D, Matsumura EE, Van Vredendaal P, Van Der Vlugt RAA. The plant virus transmissions database. J Gen Virol. 2024;105. doi:10.1099/jgv.0.001957

5. Catto MA, Mugerwa H, Myers BK, Pandey S, Dutta B, Srinivasan R. A Review on Transcriptional Responses of Interactions between Insect Vectors and Plant Viruses. Cells. 2022;11: 693. doi:10.3390/cells11040693

6. Blanc S, Drucker M, Uzest M. Localizing Viruses in Their Insect Vectors. Annu Rev Phytopathol. 2014;52: 1–23. doi:10.1146/annurev-phyto-102313-045920

7. Whitfield AE, Falk BW, Rotenberg D. Insect vector-mediated transmission of plant viruses. Virology. 2015;479–480: 278–289. doi:10.1016/j.virol.2015.03.026

8. Martín B, Collar JL, Tjallingii WF, Fereres A. Intracellular ingestion and salivation by aphids may cause the acquisition and inoculation of non-persistently transmitted plant viruses. J Gen Virol. 1997;78: 2701–2705. doi:10.1099/0022-1317-78-10-2701

9. Moreno A, Tjallingii WF, Fernandez-Mata G, Fereres A. Differences in the mechanism of inoculation between a semi-persistent and a non-persistent aphid-transmitted plant virus. J Gen Virol. 2012;93: 662–667. doi:10.1099/vir.0.037887-0

10. Powell G, Tosh CR, Hardie J. HOST PLANT SELECTION BY APHIDS: Behavioral, Evolutionary, and Applied Perspectives. Annual Review of Entomology. Annual Reviews; 2006. pp. 309–330. doi:https://doi.org/10.1146/annurev.ento.51.110104.151107

11. Blanc S, Cerutti M, Chaabihi H, Louis C, Devauchelle G, Hull R. Gene II Product of an Aphid-Nontransmissible Isolate of Cauliflower Mosaic Virus Expressed in a Baculovirus System Possesses Aphid Transmission Factor Activity. Virology. 1993;192: 651–654. doi:10.1006/viro.1993.1081

12. Uzest M, Gargani D, Drucker M, Hébrard E, Garzo E, Candresse T, et al. A protein key to plant virus transmission at the tip of the insect vector stylet. Proc Natl Acad Sci U S A. 2007;104: 17959–17964. doi:10.1073/pnas.0706608104

13. Uzest M, Gargani D, Dombrovsky A, Cazevieille C, Cot D, Blanc S. The “acrostyle”: a newly described anatomical structure in aphid stylets. Arthropod Struct Dev. 2010;39: 221–229. doi:10.1016/j.asd.2010.02.005

14. Webster CG, Pichon E, van Munster M, Monsion B, Deshoux M, Gargani D, et al. Identification of Plant Virus Receptor Candidates in the Stylets of Their Aphid Vectors. J Virol. 2018;92: e00432-18. doi:10.1128/JVI.00432-18

15. Deshoux M, Masson V, Arafah K, Voisin S, Guschinskaya N, van Munster M, et al. Cuticular Structure Proteomics in the Pea Aphid Acyrthosiphon pisum Reveals New Plant Virus Receptor Candidates at the Tip of Maxillary Stylets. J Proteome Res. 2020/01/29 ed. 2020;19: 1319–1337. doi:10.1021/acs.jproteome.9b00851

16. Rebers JE, Riddiford LM. Structure and expression of a Manduca sexta larval cuticle gene homologous to Drosophila cuticle genes. J Mol Biol. 1988;203: 411–423. doi:10.1016/0022-2836(88)90009-5

17. Deshoux M, Monsion B, Pichon E, Jiménez J, Moreno A, Cayrol B, et al. Role of Acrostyle Cuticular Proteins in the Retention of an Aphid Salivary Effector. Int J Mol Sci. 2022;23: 15337. doi:10.3390/ijms232315337

18. Le Trionnaire G, Tanguy S, Hudaverdian S, Gleonnec F, Richard G, Cayrol B, et al. An integrated protocol for targeted mutagenesis with CRISPR-Cas9 system in the pea aphid. Insect Biochem Mol Biol. 2019;110: 34–44. doi:10.1016/j.ibmb.2019.04.016

19. Cayrol B, Arnoldi I, Novak V, Epis S, Brilli M, Rahbé Y, et al. The development of the piercing mouth during the last molt of the diseases-transmitting aphids and mosquitoes as revealed by synchrotron X-ray microtomography. Entomol Gen. 2024; 105999. doi:10.1127/entomologia/2024/2475

20. Guschinskaya N, Ressnikoff D, Arafah K, Voisin S, Bulet P, Uzest M, et al. Insect Mouthpart Transcriptome Unveils Extension of Cuticular Protein Repertoire and Complex Organization. iScience. 2020;23. doi:10.1016/j.isci.2020.100828

21. Palacios I, Drucker M, Blanc S, Leite S, Moreno A, Fereres A. Cauliflower mosaic virus is preferentially acquired from the phloem by its aphid vectors. J Gen Virol. 2002;83: 3163–3171. doi:10.1099/0022-1317-83-12-3163

22. Moreno A, Palacios I, Blanc S, Fereres A. Intracellular Salivation Is the Mechanism Involved in the Inoculation of Cauliflower Mosaic Virus by Its Major Vectors <I>Brevicoryne brassicae</I> and <I>Myzus persicae</I>. Ann Entomol Soc Am. 2005;98: 763–769. doi:10.1603/0013-8746(2005)098[0763:ISITMI]2.0.CO;2

23. Walker GP, Fereres A, Tjallingii WF. Guidelines for conducting, analyzing, and interpreting electrical penetration graph (EPG) experiments on herbivorous piercing–sucking insects. Entomol Exp Appl. 2024; eea.13434. doi:10.1111/eea.13434

24. Giordanengo P. EPG-Calc: a PHP-based script to calculate electrical penetration graph (EPG) parameters. Arthropod-Plant Interact. 2014;8: 163–169. doi:10.1007/s11829-014-9298-z

25. Emms DM, Kelly S. OrthoFinder: phylogenetic orthology inference for comparative genomics. Genome Biol. 2019;20: 238. doi:10.1186/s13059-019-1832-y

26. Jousselin E, Coeur d'acier A, Clamens A-L, Galan M, Cruaud C, Barbe V, et al. Discordance between mitochondrial, nuclear, and symbiont genomes in aphid phylogenetics: who is telling the truth? Zool J Linn Soc. 2024;201: zlae098. doi:10.1093/zoolinnean/zlae098

27. Hardy NB, Kaczvinsky C, Bird G, Richter R, Dettman JR, Maw E, et al. Geographic isolation drives speciation in Nearctic aphids. Commun Biol. 2022;5: 796. doi:10.1038/s42003-022-03771-5

28. Webster CG, Thillier M, Pirolles E, Cayrol B, Blanc S, Uzest M. Proteomic composition of the acrostyle: Novel approaches to identify cuticular proteins involved in virus–insect interactions. Insect Sci. 2017;24: 990–1002. doi:10.1111/1744-7917.12469

29. Ruijter JM, Ramakers C, Hoogaars WMH, Karlen Y, Bakker O, Van Den Hoff MJB, et al. Amplification efficiency: linking baseline and bias in the analysis of quantitative PCR data. Nucleic Acids Res. 2009;37: e45–e45. doi:10.1093/nar/gkp045

30. Livak KJ, Schmittgen TD. Analysis of Relative Gene Expression Data Using Real-Time Quantitative PCR and the 2−ΔΔCT Method. Methods. 2001;25: 402–408. doi:10.1006/meth.2001.1262

31. Ryckebusch F, Peterschmitt M, Granier M, Sauvion N. Alfalfa leaf curl virus is efficiently acquired by its aphid vector Aphis craccivora but inefficiently transmitted. J Gen Virol. 2021;102. doi:10.1099/jgv.0.001516

32. Adasme-Carreño F, Muñoz-Gutiérrez C, Salinas-Cornejo J, Ramírez CC. A2EPG: A new software for the analysis of electrical penetration graphs to study plant probing behaviour of hemipteran insects. Comput Electron Agric. 2015;113: 128–135. doi:10.1016/j.compag.2015.02.005

33. Backus EA, Cline AR, Ellerseick MR, Serrano MS. <I>Lygus hesperus</I> (Hemiptera: Miridae) Feeding on Cotton: New Methods and Parameters for Analysis of Nonsequential Electrical Penetration Graph Data. Ann Entomol Soc Am. 2007;100: 296–310. doi:10.1603/0013-8746(2007)100[296:LHHMFO]2.0.CO;2

34. Mirdita M, Schütze K, Moriwaki Y, Heo L, Ovchinnikov S, Steinegger M. ColabFold: making protein folding accessible to all. Nat Methods. 2022;19: 679–682. doi:10.1038/s41592-022-01488-1

35. Sayers EW, Bolton EE, Brister JR, Canese K, Chan J, Comeau DC, et al. Database resources of the national center for biotechnology information. Nucleic Acids Res. 2022;50: D20–D26. doi:10.1093/nar/gkab1112

36. Camacho C, Coulouris G, Avagyan V, Ma N, Papadopoulos J, Bealer K, et al. BLAST+: architecture and applications. BMC Bioinformatics. 2009;10: 421. doi:10.1186/1471-2105-10-421

37. Katoh K. MAFFT: a novel method for rapid multiple sequence alignment based on fast Fourier transform. Nucleic Acids Res. 2002;30: 3059–3066. doi:10.1093/nar/gkf436

38. Katoh K, Standley DM. MAFFT Multiple Sequence Alignment Software Version 7: Improvements in Performance and Usability. Mol Biol Evol. 2013;30: 772–780. doi:10.1093/molbev/mst010

39. Minh BQ, Schmidt HA, Chernomor O, Schrempf D, Woodhams MD, Von Haeseler A, et al. IQ-TREE 2: New Models and Efficient Methods for Phylogenetic Inference in the Genomic Era. Teeling E, editor. Mol Biol Evol. 2020;37: 1530–1534. doi:10.1093/molbev/msaa015

40. R Core Team (2020). R Foundation for Statistical Computing, Vienna, Austria.; Available: https://www.R-project.org/

Click here to access/download
Supporting Information
Fu-et-al_Supporting_Information.pdf
