# Supplementary material for: Stylet cuticular gene-directed mutagenesis impairs the pea aphid vector capacity to transmit a plant virus
Source: PLoS Pathog. 2025 May 23;21(5):e1013192. doi: 10.1371/journal.ppat.1013192 (PMC12140417; doi:10.1371/journal.ppat.1013192)
Supplement: Attachment — Submitted filename: PPATHOGENS-D-25-00024_Response_to_reviewers.docx [file ppat.1013192.s016.docx]

Response to Reviewers

PPATHOGENS-D-25-00024
Stylet cuticular gene-directed mutagenesis impairs the pea aphid vector capacity to transmit a plant virus

Dear Editors,

We want to express our sincere gratitude to the reviewers for their appreciation of our work and the editors and reviewers for the opportunity to clarify specific points, which has helped us improve the manuscript. A point-by-point answer is provided in the text below.

------------

**Journal Requirements:**

1) We ask that a manuscript source file is provided at Revision. Please upload your manuscript file as a .doc, .docx, .rtf or .tex. If you are providing a .tex file, please upload it under the item type u2018LaTeX Source Fileu2019 and leave your .pdf version as the item type u2018Manuscriptu2019.

2) We do not publish any copyright or trademark symbols that usually accompany proprietary names, eg ©,  ®, or TM  (e.g. next to drug or reagent names). Therefore please remove all instances of trademark/copyright symbols throughout the text, including:
- ® on pages: 24 and 27.

As requested, we removed all trademark/copyright symbols.

3) Please upload all main figures as separate Figure files in .tif or .eps format. For more information about how to convert and format your figure files please see our guidelines: 
<https://journals.plos.org/plospathogens/s/figures>

4) We have noticed that you have uploaded Supporting Information files, but you have not included a list of legends. Please add a full list of legends for your Supporting Information files after the references list.

We added the list of legends of Supporting Information files after the list of references.

5) Some material included in your submission may be copyrighted. According to PLOSu2019s copyright policy, authors who use figures or other material (e.g., graphics, clipart, maps) from another author or copyright holder must demonstrate or obtain permission to publish this material under the Creative Commons Attribution 4.0 International (CC BY 4.0) License used by PLOS journals. Please closely review the details of PLOSu2019s copyright requirements here: PLOS Licenses and Copyright. If you need to request permissions from a copyright holder, you may use PLOS's Copyright Content Permission form.
Please respond directly to this email and provide any known details concerning your material's license terms and permissions required for reuse, even if you have not yet obtained copyright permissions or are unsure of your material's copyright compatibility. Once you have responded and addressed all other outstanding technical requirements, you may resubmit your manuscript within Editorial Manager. 
Potential Copyright Issues:

- Please confirm (a) that you are the photographer of Figure 1A, and 5, or (b) provide written permission from the photographer to publish the photo(s) under our CC BY 4.0 license.

Yu Fu, the first author of the paper, took the images of aphids Figure 1A, specifically to illustrate the work presented in this manuscript. We specified the credit in the legend.

All microscopy images of aphid stylets presented in this manuscript figures were acquired for the present work by the authors of this manuscript.

6) Please amend your detailed Financial Disclosure statement. This is published with the article. It must therefore be completed in full sentences and contain the exact wording you wish to be published. Please ensure that the funders and grant numbers match between the Financial Disclosure field and the Funding Information tab in your submission form. Note that the funders must be provided in the same order in both places as well. State the initials, alongside each funding source, of each author to receive each grant. For example: "This work was supported by the National Institutes of Health (####### to AM; ###### to CJ) and the National Science Foundation (###### to AM).".
If you did not receive any funding for this study, please simply state: u201cThe authors received no specific funding for this work.u201d

We updated the funding description and acknowledgements sections as follows and included them in the manuscript:

“**Fundings**

This work was funded by the French National Research Agency (ANR-15-CE20-0011 to MU ; and ANR-21-CE20-0001 to MU), the Bill and Melinda Gates Foundation (GCEag, OPP1130147 to MU), the StylED project (défi clé RIVOC Occitanie Region, Université of Montpellier to EJ and SC). Yu Fu acknowledges the Chinese Scholarship Council (CSC Grant No. 202103250004) and the French National Institute Research for Agriculture, Food and Environment INRAE, for financial support of her PhD studies. The funders had no role in the study design, data collection and analysis, decision to publish, or preparation of the manuscript.

**Acknowlegments**

All rearing and experiments on insects have been performed on the Baillarguet Vectoplante platform, member of the Vectopole Sud Network (<http://www.vectopole-sud.fr/>).”

------------

**Reviewers' Comments:**

**Part II – Major Issues: Key Experiments Required for Acceptance**

Reviewer #1: The raw data for the figures should be made available via a data repository, or provided as a supplemental spreadsheet / document. This is particularly true given the nature of statistical packages used for the analysis.

We have compiled all relevant raw data corresponding to figures 2, 3B, 6, 7B, 7D in a summary Excel File entitled 'Supporting Information 2', including a summary table of contents.

Reviewer #2: 1. In lines 119-121, the authors mention that the Sty01-Cter line lacks the 11 C-terminal amino acids that form the surface-exposed domain of the acrostyle, which is thought to interact with viruses. Did the researchers test whether this domain is essential for binding to CaMV? To properly verify this interaction, co-immunoprecipitation (Co-IP) or immunofluorescence co-localization experiments should have been conducted.

We agree with Reviewer #2 that a direct interaction between stylin-derived peptides and the viral protein P2 would help define the Stylin-01 motif essential for P2 binding. We have developed many approaches and constructs over the past few years to address this question both *in vivo* (in bacteria, insect cells, yeast, and plants) and *in vitro* (including Far western-blot, co-IP, GST pull-down, GFP-Trap, peptide arrays, phage display). Despite sustained efforts, all our attempts failed up to now. We strongly suspect that these cuticular proteins lose their binding activity out of their chitin context, and the same is probably true for derived peptides.

A paragraph has been added in the Discussion section of the revised version to discuss these points:

“Only the last 11 C-terminal amino acids from the extended 33 AA C-terminal domain in the Sty01-Cter mutant are missing in allele 1 and are different in allele 2 compared to the wild type. Based on the 3D AlphaFold prediction in both alleles, the exposed helix structure is conserved (Fig 1). Whether the helix itself or the whole domain is important for P2 binding would need further investigation. But unfortunately, interactions between the viral protein P2 and cuticular protein derived-peptides cannot be tested with classical approaches [14,28].”

2. Fig 3 demonstrates a reduction in the number of positively labeled stylets for both mutant lines compared to WT aphids. However, it appears that a significant majority of the mutant aphids (over 80%) still retain the ability to bind CaMV P2 proteins and thus can transmit the virus. This raises the question of whether Stylin-01 is indeed the most critical protein regulating viral transmission. While the gene knockout has a statistically significant effect, the impact on virus transmission does not seem substantial enough to conclusively establish Stylin-01 as the primary regulator. This observation suggests that other factors or proteins might also play crucial roles in CaMV transmission, which deserves discussion.

Even if “a significant majority of the mutant aphids (over 80%) still retain the ability to bind CaMV P2 proteins” our data demonstrate that the mutants cannot transmit the virus as efficiently as the wild-type. This support the crucial role of Stylin-01 as a receptor of the plant virus CaMV. Collectively our paper shows that Stylin-01 is a primary actor in CaMV transmission, albeit not being the only as discussed in the following updated paragraph:

“We show here that the viral protein P2 can still bind to the maxillary stylets of both mutant aphids even if with less efficiency, indicating that Stylin-01 is not the sole acrostyle protein with CaMV-binding activities. Disrupting *stylin-01* does not change the overall stylet ultrastructure but affects the surface distribution of other stylins.The localization of Stylin-02 on the surface of the Sty01-KO mutant stylets is compatible with the localization of CaMV P2 binding sites, while that of Stylin-03 or Stylin-04/04bis is not (Fig 5). The high similarity between Stylin-01 and -02 in their exposed domains prevents the development of specific antibodies. The antibody recognizing both putative orthologs showed no difference in acrostyle labeling between mutants and wild-type lines (Fig 5). Differences in the labeling of mutant lines could have been expected, particularly in the Sty01-KO one where only Stylin-02 is present. The fully labeled acrostyle in the KO mutant suggests that Stylin-02 may compensate for the missing Stylin-01 protein in organizing this cuticular region that is essential for plant-aphid interactions. This potential role is further supported by Stylin-02 overexpression during stylet biogenesis in the mutant lines and by evolutionary analyses that indicate that Stylin-01 and Stylin-02 are orthologs. Nonetheless, Stylin-02 distribution, even if aligned with P2 binding sites does not seem sufficient to complement the lack of Stylin-01 restoring CaMV transmission in the KO mutant.”

Reviewer #3: (No Response)

---------

**Part III – Minor Issues: Editorial and Data Presentation Modifications**

Reviewer #1: The writing style is at times cumbersome with excessive use of comas and prepositions. The PDF is provided with several dozen suggested edits.

We included all suggested modifications in the new text and tried to simplify it to respond to this suggestion.

The presentation of statistical includes non-parametric tests associated with non-normal distribution. Additional detail, including the assessment of normality would seem appropriate, even though the work understandably has limited numbers (making normality tests problematic). This statistical analysis seems to rely on the authors use of packages, more so than an understanding of the underlying statistical tests.

Thanks to this remark, we realized that the original presentation of the statistical methods section could give the incorrect impression that we had chosen the tools rather than the appropriate test. Indeed, we chose the most appropriate statistical tests based on the size of the datasets to be analyzed. We revised the Material and Methods section to clarify this point by moving the tools citation to the end of the paragraph as follows:

“Statistical analysis were performed using tests adapted to each data set. Differences in aphid transmission efficiencies were tested using a binomial generalized linear model (GLM) followed by a post-hoc Tukey's honestly significant difference (HSD) test. Differences in stylets labeling were analyzed by GLM. The qPCR data were analyzed using a one-way analysis of variance (ANOVA), followed by the least significant difference (LSD) test. Differences in EPG variables and the duration of nymph development were analyzed using Kruskal-Wallis test. Different letters indicate significant differences among groups, and asterisks denoted statistical significance between two groups (*P < 0.05, **P < 0.01, and ***P < 0.001). All the data were analyzed using SPSS Statistics 20.0 software or R Stats Package [40], and plotted using GraphPad Prism version 9.”

The discussion is quite redundant with the results, including reiterated references to the figures rather than extending the analysis to a broader perspective. This section could be considerably shortened.

We modified the Discussion section to avoid redundancy.

The effort made in high quality presentation of graphics and tables (including supplemental) is noted. The overall quality and quantity of research represents a excellent addition to the literature.

Reviewer #2: 1. The antibodies used for western blotting and immunofluorescent localization, particularly those against Sty01-Cter and Sty01-KO in Fig 1, 3, and 5, should be clearly indicated either in the figures themselves or thoroughly described in the figure legends. This will help readers better understand and interpret the data presented in the figures.

We specified the antibodies used in Figures 1 and 5, as well as in their respective legends. Following Reviewer #2's recommendation, we have completed and clarified this information to make it easier to understand the figures and the legends. Regarding Figure 3, we used no antibody: the green fluorescence corresponds to a GFP-fused protein. We explicitly stated this in the figure legend following the reviewer's observation.

2. Fig 3B presents the number of stylets observed for each group from a single experiment batch. To establish the reproducibility of these results, it is a standard practice to conduct at least 3 independent experimental batches. Without multiple batches, the results may lack statistical significance and robustness. It is crucial to know whether the authors conducted different experimental batches to validate their findings.

Indeed, we performed 3-4 independent experiments, as the reviewer noted. The data are in fact, combined in the graph of Figure 3B. We explained the design in the text/legend, and we now provide the raw data in Supporting Information 2.

Reviewer #3: (No Response)
